# An Alternative σ Factor, σ^8^, Controls Avermectin Production and Multiple Stress Responses in *Streptomyces avermitilis*

**DOI:** 10.3389/fmicb.2017.00736

**Published:** 2017-04-24

**Authors:** Di Sun, Qian Wang, Zhi Chen, Jilun Li, Ying Wen

**Affiliations:** State Key Laboratory of Agrobiotechnology and MOA Key Laboratory of Soil Microbiology, College of Biological Sciences, China Agricultural UniversityBeijing, China

**Keywords:** *Streptomyces avermitilis*, avermectins, alternative σ factor, σ^8^, stress response

## Abstract

Alternative σ factors in bacteria redirect RNA polymerase to recognize alternative promoters, thereby facilitating coordinated gene expression necessary for adaptive responses. The gene *sig8* (*sav_741*) in *Streptomyces avermitilis* encodes an alternative σ factor, σ^8^, highly homologous to σ^B^ in *Streptomyces coelicolor*. Studies reported here demonstrate that σ^8^ is an important regulator of both avermectin production and stress responses in *S. avermitilis*. σ^8^ inhibited avermectin production by indirectly repressing expression of cluster-situated activator gene *aveR*, and by directly initiating transcription of its downstream gene *sav_742*, which encodes a direct repressor of *ave* structural genes. σ^8^ had no effect on cell growth or morphological differentiation under normal growth conditions. Growth of a *sig8-*deletion mutant was less than that of wild-type strain on YMS plates following treatment with heat, H_2_O_2_, diamide, NaCl, or KCl. *sig8* transcription was strongly induced by these environmental stresses, indicating response by σ^8^ itself. A series of σ^8^-dependent genes responsive to heat, oxidative and osmotic stress were identified by EMSAs, qRT-PCR and *in vitro* transcription experiments. These findings indicate that σ^8^ plays an important role in mediating protective responses to various stress conditions by activating transcription of its target genes. Six σ^8^-binding promoter sequences were determined and consensus binding sequence BGVNVH-N_15_-GSNNHH (B: C, T or G, V: A, C or G, S: C or G, H: A, C or T, N: any nucleotide) was identified, leading to prediction of the σ^8^ regulon. The list consists of 940 putative σ^8^ target genes, assignable to 17 functional groups, suggesting the wide range of cellular functions controlled by σ^8^ in *S. avermitilis*.

## Introduction

Soil-inhabiting filamentous *Streptomyces* are characterized by the ability to produce valuable secondary metabolites having antimicrobial, anticancer, anthelmintic, immunosuppressive, or other biological activities (Challis and Hopwood, 2003). Biosynthesis of secondary metabolites is usually accompanied by initiation of morphological differentiation, and precisely controlled by complex regulatory networks involving cluster-situated regulators (CSRs) and higher-level global/pleiotropic regulators in response to various environmental and endogenous signals (van Wezel and McDowall, 2011; Liu et al., 2013; Niu et al., 2016; Urem et al., 2016). Elucidation of these regulatory networks is essential for strain improvement by metabolic engineering approaches.

*Streptomyces avermitilis* is an important industrial microorganism used for production of avermectins, effective anthelmintic agents widely applied in agricultural and medical fields (Burg et al., 1979; Egerton et al., 1979). *aveR*, located at the left edge of the *ave* gene cluster, encodes a LuxR-family cluster-situated activator (Kitani et al., 2009; Guo et al., 2010). The *aveR* promoter is recognized by a housekeeping σ factor, σ^hrdB^ (Zhuo et al., 2010). We showed that two extracytoplasmic function (ECF) σ factors, σ^6^ (SAV663) and σ^25^ (SAV3351), inhibit avermectin production by indirectly affecting *aveR* transcription (Jiang et al., 2011; Luo et al., 2014). The other 57 σ factors in *S. avermitilis* remain to be characterized. Elucidation of their functions will help to clarify the regulatory networks involved in avermectin biosynthesis.

With very few exceptions, bacterial σ factors belong to the σ^70^ family, which is divided into four groups (Groups 1–4) based on differential possession of four conserved domains (σ_1_, σ_2_, σ_3_, and σ_4_), phylogenic relationships and physical functions (Osterberg et al., 2011). Group 1 housekeeping sigma factors possess all four domains and are necessary for growth. Group 2 sigma factors also contain four domains, but are dispensable for growth. Group 3 alternative σ factors lack a σ_1_ domain and are involved mainly in stress response and differentiation processes. Group 4 ECF sigma factors contain only σ_2_ and σ_4_ domains and usually respond to extracytoplasmic stimuli. The first identified alternative σ factor is σ^B^ in *Bacillus subtilis* (Haldenwang and Losick, 1980), which functions as a master regulator that controls >200 genes in response to a wide variety of stress/starvation stimuli including glucose, phosphate, or oxygen starvation, heat or cold shock, ethanol, acid, or osmotic stress, nitric oxide, and antibiotic-induced cell wall damage (Lee et al., 2005; Hecker et al., 2007). *sigB* and its homologes are widespread among Gram-positive bacteria, and have diverse functions (Hecker et al., 2007). SCO0600, the σ^B^ homolog in *Streptomyces coelicolor*, responds to osmotic stress, but not to heat, ethanol, or H_2_O_2_ stress (Cho et al., 2001; Lee et al., 2004a, 2005). σ^B^ is also involved in secondary metabolism and morphological differentiation in *S. coelicolor*. The *sigB* deletion mutant of this species displayed overproduction of actinorhodin (ACT), reduced production of undecylprodigosin (RED) and lack of aerial mycelium formation on R2YE or NA plates (Cho et al., 2001). In *Streptomyces hygroscopicus* 5008, *sigB* transcription was enhanced by heat stress or treatment with a reactive oxygen species (ROS) inhibitor, suggesting involvement of σ^B^ in response to changes in temperature or ROS level (Wei et al., 2012).

We demonstrated recently that SAV742, a novel AraC-family transcriptional regulator in *S. avermitilis*, directly represses *ave* structural genes and controls cell growth and morphological differentiation (Sun et al., 2016). The gene adjacent to *sav_742*, *sav_741* (*sig8*), encodes an alternative σ factor, σ^8^, homologous to σ^B^ in *S. coelicolor*. We describe here characterization of σ^8^ as an important regulator that controls avermectin biosynthesis and multiple stress responses in *S. avermitilis*. σ^8^ represses avermectin biosynthesis in part through direct initiation of *sav_742* transcription. Unlike its *S. coelicolor* homolog σ^B^, σ^8^ responds to heat, osmotic and oxidative stress by directly regulating expression of its own gene and certain other stress protection genes, but is not involved in morphological differentiation or cell growth. Moreover, we predicted the σ^8^ regulon based on the consensus σ^8^-binding promoter sequence.

## Materials and Methods

### Primers, Plasmids, Strains, and Growth Conditions

The strains and plasmids used or constructed in this study are listed in **Table 1**, and the primers in Supplementary Table S1. Culture conditions for *Escherichia coli* and *S. avermitilis* strains were as described previously (Liu W. et al., 2015). MM, R2YE (Kieser et al., 2000) and YMS (Ikeda et al., 1988) plates were used for phenotypic observation of *S. avermitilis* strains. Insoluble fermentation medium FM-I (Chen et al., 2007) was used for routine avermectin production. Soluble fermentation medium FM-II (Guo et al., 2010) was used to grow mycelia for biomass analysis, and for RNA isolation following stress treatment.

**Table 1:** Strains and plasmids used in this study.

Strain or plasmid	Description	Source or reference
**Strains**		
*Streptomyces avermitilis*		
ATCC31267	Wild-type (WT) strain, a natural avermectin producer	Laboratory stock
Dsig8	*sig8* deletion mutant	This study
Csig8	*sig8* complemented strain	This study
Osig8	*sig8* overexpression strain	This study
D742	*sav_742* deletion mutant	Sun et al., 2016
Dsig8–742	*sig8 sav_742* double deletion mutant	This study
WT/pKC1139	WT strain carrying control vector pKC1139	This study
WT/pSET152	WT strain carrying control vector pSET152	This study
*Escherichia coli*		
JM109	Routine cloning host	Laboratory stock
ET12567	*dam dcm hsdS cat tet*, non-methylating strain	Macneil and Klapko, 1987
BL21 (DE3)	Protein expression host	Novagen
**Plasmids**		
pKC1139	Temperature-sensitive multiple-copy *E. coli -Streptomyces* shuttle vector for gene deletion or overexpression in *S. avermitilis*	Bierman et al., 1992
pSET152	Integrative vector for integrating a single-copy gene into the *S. avermitilis* chromosome	Bierman et al., 1992
pET-28a (+)	Protein expression vector	Novagen
pJL117	pIJ2925 derivative with insertion of *ermE^∗^p* (*Streptomyces* strong constitutive promoter)	Li et al., 2010
pJL117-sig8	pJL117 carrying *sig8* ORF	This study
pKCDsig8	*sig8* deletion vector based on pKC1139	This study
pSET152-sig8	*sig8* complemented vector based on pSET152	This study
pKC1139-erm-sig8	*sig8* overexpression vector based on pKC1139	This study
pET-sig8	*sig8* overexpression vector based on pET-28a (+)	This study


### Gene Deletion, Complementation, and Overexpression

A *sig8* in-frame gene deletion mutant was constructed using a homologous recombination strategy. Two homologous fragments flanking *sig8* were amplified by PCR from wild-type (WT) genomic DNA. A 529-bp 5′-flanking region (positions -337 to +192 relative to the *sig8* start codon) was amplified with primers SD2A/SD2B, and a 541-bp 3′-flanking region (positions +835 to +1375) was amplified with primers SD3A/SD3B. The two fragments were cut with *Hin*dIII/*Bam*HI and *Bam*HI/*Eco*RI, respectively, and then simultaneously cloned into *Hin*dIII/*Eco*RI-digested pKC1139 to generate *sig8* deletion vector pKCDsig8. Non-methylated pKCDsig8 was transformed into WT protoplasts, and double-crossover recombinant strains were isolated as reported previously (Zhao et al., 2007). Resulting *sig8* deletion mutants were verified by colony PCR using primers SD23A/SD23B (flanking the exchange regions) and SD39A/SD39B (located within the deletion region of *sig8*) (Supplementary Figure S1), followed by DNA sequencing. When primers SD23A/SD23B were used, a 1.2-kb band appeared, whereas a 1.9-kb band was detected in WT genomic DNA. When primers SD39A/SD39B were used, only WT DNA generated a 390-bp band. We thus obtained *sig8* gene deletion mutant Dsig8, in which 840-bp *sig8* ORF was mostly deleted (from positions +193 to +834 relative to the start codon) (Supplementary Figure S1). The deletion part of *sig8* covered coding region for all three functional domains (σ_2_, σ_3_, and σ_4_); Thus, the remaining fragment was unlikely to be functional.

To construct a *sig8sav_742* double deletion mutant, the pKCDsig8 vector was transformed into D742 protoplasts (Sun et al., 2016). The expected mutant, Dsig8–742, was isolated by selection of the Dsig8 mutant.

For complementation of Dsig8, a 1618-bp PCR fragment containing the *sig8* promoter and open reading frame (ORF) was amplified with primers SD1B/SD1D, and inserted into the integrative vector pSET152 to give *sig8*-complemented vector pSET152-sig8, which was then transformed into Dsig8 to obtain complemented strain Csig8.

For overexpression of *sig8*, a 987-bp fragment carrying the *sig8* ORF was amplified using primers SD1B/SD1C, and inserted into pJL117 to generate pJL117-sig8, in which *sig8* was controlled by *ermE^∗^p* (*Streptomyces* strong constitutive promoter). The 1.3-kb *Eco*RI/*Hin*dIII fragment containing *ermE^∗^p* and *sig8* ORF from pJL117-sig8 was ligated into pKC1139 to generate *sig8* overexpression vector pKC1139-erm-sig8, which was transformed into WT protoplast to construct *sig8* overexpression strain Osig8.

### Production and Analysis of Avermectins

Fermentation of *S. avermitilis* strains and HPLC (high-pressure liquid chromatography) analysis of avermectin yield in fermentation broth were performed as described previously (Chen et al., 2007).

### Quantitative Real-Time RT-PCR (qRT-PCR)

*S. avermitilis* mycelia grown in FM-I or FM-II were collected at various time points for RNA isolation. Triturated samples were suspended in TRIzol reagent (Tiangen, China) for RNA isolation. Genomic DNA contamination was removed by treatment of RNA samples with RNase-free DNase I (TaKaRa, China). 4 μg total RNA was used for cDNA synthesis. qRT-PCR was performed using primers listed in Supplementary Table S1 to analyze transcription levels of the tested genes as described previously (Luo et al., 2014), with expression level of housekeeping gene 16S *rRNA* as internal control. Each experiment was repeated three times.

### Overexpression and Renaturation of His_6_-σ^8^

The 953-bp *sig8* coding region (279 amino acids) was amplified from WT genomic DNA using primers SD1A/SD1B. The obtained PCR product was inserted into pET-28a (+) to generate pET-sig8. After confirmation by DNA sequencing, pET-sig8 was transformed into *E. coli* BL21 (DE3) for His_6_-σ^8^ (N-terminal His_6_-tagged σ^8^ recombinant protein) overexpression. Following IPTG induction, cells containing His_6_-σ^8^ were harvested, washed, resuspended in a lysis buffer (Luo et al., 2014), and disrupted by sonication on ice. His_6_-σ^8^ present as inclusion body in *E. coli* was purified, solubilized, and renatured as described previously (Luo et al., 2014). Purified renatured soluble His_6_-σ^8^ was quantified and stored at -80°C for use *in vitro* transcription experiments and electrophoretic mobility shift assays (EMSAs).

### Electrophoretic Mobility Shift Assays (EMSAs)

EMSA probes carrying respective tested promoter regions were obtained by PCR using primers listed in Supplementary Table S1, and labeled at their 3′ ends with digoxigenin (DIG). Conditions for binding reaction and detection were as described previously (Sun et al., 2016). To confirm binding specificity between His_6_-σ^8^ and DNA probes, a ∼300-fold excess of each specific or non-specific (*hrdB*) unlabeled probe was added to the reaction mixture before incubation.

### *In Vitro* Transcription Assay

DNA templates containing respective promoter and partial coding region were amplified by PCR using primers listed in Supplementary Table S1. Conditions for transcription assays and detection of transcripts were as described previously (Luo et al., 2014). Reaction mixtures contained 0.4 pmol DNA template and various amounts of renatured His_6_-σ^8^. Transcription was initiated by addition of 3.7 pmol *E. coli* core RNA polymerase (core-RNAP) (New England Biolabs, USA).

### 5′ Rapid Amplification of cDNA Ends (5′ RACE)

The transcriptional start site (TSS) of selected genes was identified using a 5′/3′ RACE kit (Roche, 2nd Generation). *S. avermitilis* strains were cultured in FM-II at 28°C for 2 days, and then subjected to various stress conditions. Mycelia were harvested after various durations of stress treatment and used for RNA isolation. 4 μg total RNA was reverse transcribed with 40 pmol gene-specific primer SP1. Purified cDNAs were added to oligo-dA tails at the 3′ end by TdT (terminal deoxynucleotidyl transferase, TaKaRa) treatment at 37°C for 30 min. The tailed cDNA was used as template for first-round PCR using second inner gene-specific primer SP2 and oligo-dT anchor primer. The resulting PCR product was diluted to appropriate concentration, and used as template for second-round PCR with nested primer SP3 and an anchor primer. The purified final PCR product was sequenced (Invitrogen Biotechnology Corporation, China). TSS was determined as the first nucleotide following the oligo-dT sequence.

## Results

### σ^8^ Regulates Avermectin Production by Repressing *ave* Gene Expression during the Late Fermentation Stage

**FIGURE 1:**
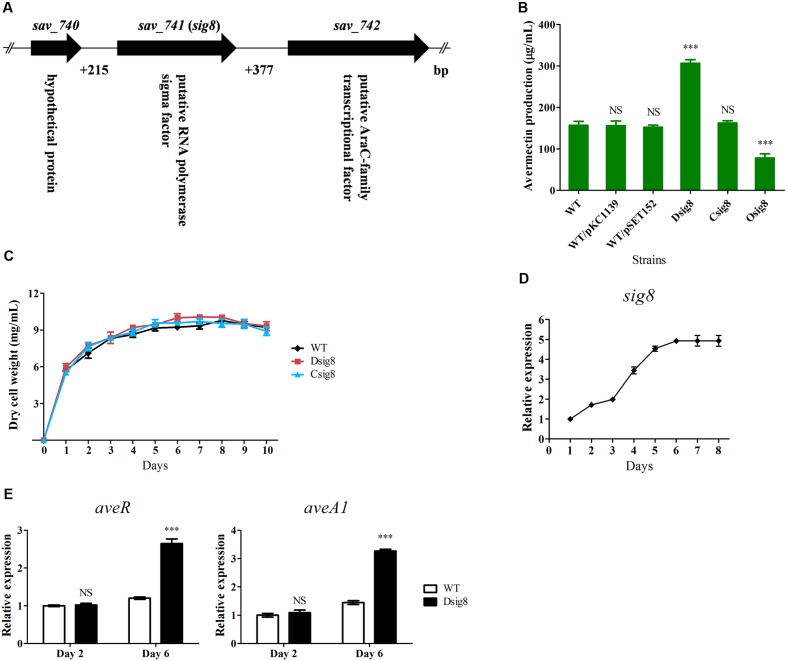
**Effects of σ^8^ on avermectin production, cell growth and *ave* gene transcription of *S. avermitilis*.** **(A)** Schematic diagram of *sig8* and its neighboring genes. **(B)** Comparative avermectin yield in WT, *sig8* deletion mutant (Dsig8), complemented strain (Csig8) and overexpression strain (Osig8) grown in FM-I for 10 days. WT/pKC1139 and WT/pSET152: vector control strains constructed by transformation of plasmids pKC1139 and pSET152 into WT, respectively. Error bar: SD from three independent experiments. NS, not significant; ^∗∗∗^, *P* < 0.001 for comparison of values for mutant versus WT strains (Student’s *t*-test). **(C)** Growth curves of WT, Dsig8 and Csig8 in soluble FM-II. **(D)** Transcriptional profile of *sig8* in WT grown in FM-I. Values were calculated by normalization against internal control gene 16S *rRNA* at specific time points. The relative value of *sig8* at day 1 was assigned as 1. **(E)** qRT-PCR analysis of *aveR* and *aveA1* from WT and Dsig8 grown in FM-I at days 2 and 6. Transcription level of each gene was expressed relative to WT value at day 2, defined as 1. NS, not significant; ^∗∗∗^, *P* < 0.001 (Student’s *t*-test).

The *sig8* (*sav_741*) gene in the *S. avermitilis* chromosome contains 840 nucleotides (nt) and encodes a 279-amino-acid σ^70^-family alternative σ factor, σ^8^. The downstream convergently transcribed gene *sav_742*, located 377 nt from *sig8* (**Figure 1A**), encodes an AraC-family transcriptional regulator that was recently characterized as a global regulator of avermectin biosynthesis, cell growth, and morphological development (Sun et al., 2016). The upstream convergently transcribed gene *sav_740*, located 215 nt from *sig8*, encodes a hypothetical protein. BLAST analysis revealed that σ^8^ displays high amino acid sequence identities with *S. coelicolor* σ^B^ (77.22%) and *S. hygroscopicus* σ^B^ (78.29%).

To investigate the role of σ^8^ in avermectin biosynthesis, we constructed *sig8* deletion mutant Dsig8, complemented strain Csig8 and overexpression strain Osig8, and compared their avermectin yields with that of WT ATCC31267 cultured in FM-I for 10 days. Relative to WT yield, that of Dsig8 was ∼96% higher, that of Osig8 was ∼50% lower, and that of Csig8 was similar (**Figure 1B**). Yields of two vector control strains, WT/pSET152 and WT/pKC1139, were almost the same as that of WT (**Figure 1B**). These findings indicate that σ^8^ has a negative effect on avermectin production.

To assess the effect of σ^8^ on *S. avermitilis* growth, we measured biomasses of WT, Dsig8 and Csig8 cultured in soluble FM-II. Biomass of Dsig8 and Csig8 was similar to that of WT (**Figure 1C**), indicating that σ^8^ has no effect on cell growth, and that avermectin overproduction in Dsig8 does not result from alteration of growth. Dsig8 and Osig8 grew normally on YMS, R2YE, and MM plates (Supplementary Figure S2), indicating that σ^8^ also has no effect on morphological differentiation.

To investigate the relationship between σ^8^ and avermectin production, we examined the *sig8* transcription profile by qRT-PCR using RNAs isolated from WT in FM-I. *sig8* transcript was detected throughout the fermentation process. Its level increased gradually from day 1, reached a maximum on day 6, and remained high thereafter, suggesting that σ^8^ plays its regulatory role mainly during the late stage of fermentation (**Figure 1D**).

The effect of σ^8^ on expression of *ave* genes was assessed by qRT-PCR analysis of transcription levels of *aveR* (CSR gene) and *aveA1* (structural gene encoding AVES1 polyketide synthase) in WT and Dsig8 grown in FM-I for 2 days (early exponential phase) or 6 days (stationary phase). Transcription levels of *aveR* and *aveA1* in Dsig8 did not differ from those in WT on day 2, but were notably higher on day 6 (**Figure 1E**), consistent with the observed avermectin overproduction in Dsig8. These findings indicate that σ^8^ controls avermectin production at the transcription level by repressing *ave* genes, primarily in the late fermentation stage.

### σ^8^ Directly Activates Expression of Its Own Gene and *sav_742*, Indirectly Regulates *ave* Genes

**FIGURE 2:**
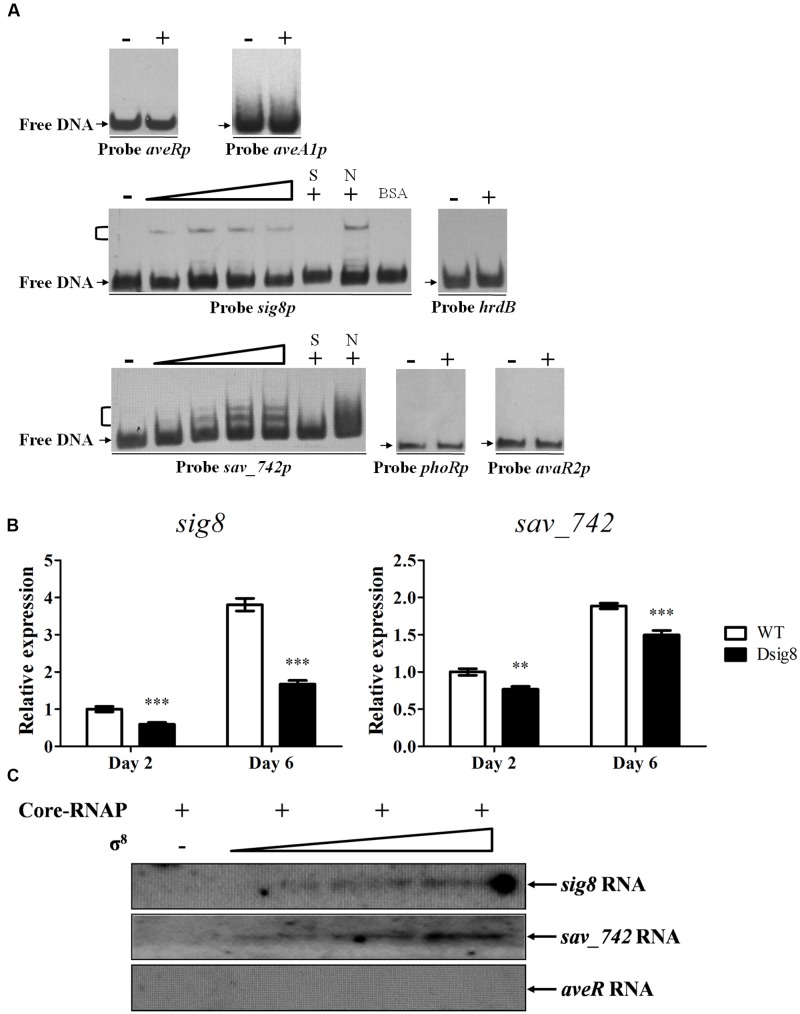
**Initiation of *sig8* and *sav_742* transcription by σ^8^.** **(A)** EMSAs of His_6_-σ^8^ interaction with probes *aveRp*, *aveA1p*, *sig8p*, *sav_742p*, *phoRp*, and *avaR2p*. Each lane contained 0.15 nM labeled probe. For competition assays, ∼300-fold excess of unlabeled specific (lanes S) or non-specific (lanes N) competitor DNA was used. Lanes –: EMSAs without His_6_-σ^8^. Lanes 2 to 5, for probes *sig8p* and *sav_742p*, contained respectively 0.25, 0.5, 0.75, and 1 μM renatured His_6_-σ^8^. 1 μM His_6_-σ^8^ was used for competition assays and probes *aveRp*, *aveA1p*, *phoRp*, *avaR2p*, and *hrdB* (lanes +). Negative probe: labeled non-specific probe *hrdB*. Negative protein control: 0.1% BSA. Arrows: free probes. Brackets: σ^8^-DNA complexes. **(B)** qRT-PCR analysis of *sig8* and *sav_742* in WT and Dsig8 grown in FM-I. Transcription level of each gene is expressed relative to WT value at day 2, defined as 1. ^∗∗^, *P* < 0.01; ^∗∗∗^, *P* < 0.001 (*t-*test). **(C)** *In vitro* transcription assay of *sig8*, *sav_742* and *aveR* promoters by RNA polymerase containing σ^8^ (Eσ^8^). Each reaction mixture contained 0.4 pmol DNA template. Eσ^8^ holoenzyme was reconstituted by mixing 3.7 pmol *Escherichia coli* core-RNAP with various amounts (30, 70, 110 pmol) of renatured His_6_-σ^8^.

σ factors function as initiators of transcription. The negative regulatory effects of σ^8^ on *aveR* and *aveA1* are therefore likely to be indirect. To test this idea, we performed EMSAs using refolded soluble recombinant His_6_-σ^8^ and probes *aveRp* and *aveA1p* prepared by labeling promoter regions of *aveR* and *aveA1*. His_6_-σ^8^ did not bind to the probes even at high concentration (1 μM) (**Figure 2A**), confirming that σ^8^ indirectly represses *ave* genes.

σ^B^ homologes typically initiate their own transcription (Hecker et al., 2007). The finding that σ^8^ indirectly represses *ave* genes suggested that σ^8^ initiates transcription of direct repressor(s) of *aveR* or *aveA1*. We recently characterized PhoP (Yang et al., 2015) and AvaR2 (Zhu et al., 2016) as direct repressors of *aveR*. We also found that SAV742 directly represses transcription of several *ave* structural genes including *aveA1*, but not *aveR* (Sun et al., 2016). To test the hypothesis that σ^8^ directly controls *sig8* and regulatory genes *phoP*, *avaR2* and *sav_742*, we performed EMSAs, qRT-PCR analysis and *in vitro* transcription assays.

EMSA results revealed that His_6_-σ^8^ formed complexes with promoter regions of *sig8* (probe *sig8p*) and *sav_742* (probe *sav_742p*), but not with those of *avaR2* (probe *avaR2p*) or *phoR-phoP* operon (probe *phoRp*) (**Figure 2A**). No shifted band was observed for negative probe control *hrdB* or protein control BSA. Binding specificity was confirmed by competitive assays using excess unlabeled specific probe (lanes S) and non-specific probe *hrdB* (lanes N). qRT-PCR analysis showed that transcription levels of *sig8* and *sav_742* on days 2 and 6 were lower in Dsig8 than in WT (**Figure 2B**), indicating that σ^8^ positively regulates expression of its own gene and adjacent gene *sav_742*. The lower *sav_742* transcription level and higher avermectin yield in Dsig8 are consistent with SAV742’s function as a repressor of avermectin production (Sun et al., 2016).

*In vitro* transcription experiments were performed to determine whether σ^8^ initiates transcription of *sig8* and *sav_742*. Linear DNA fragments harboring *sig8*, *sav_742*, and *aveR* promoter regions were used as templates, and various amounts of renatured His_6_-σ^8^ were mixed with sufficient *E. coli* core-RNAP to reconstitute RNAP holoenzyme (Eσ^8^). Core-RNAP alone did not initiate transcription at the *sig8* or *sav_742* promoter, whereas the Eσ^8^ holoenzyme did (**Figure 2C**). When core-RNAP concentration was kept constant and σ^8^ concentration was increased, transcript levels of *sig8* and *sav_742* increased, but no *aveR* transcript was detected. These findings indicate that σ^8^ initiates transcription of its own gene and *sav_742*, but not that of *aveR*. The presence of *sig8* and *sav_742* transcripts in Dsig8 suggests that additional σ factors may recognize *sig8* and *sav_742* promoters and initiate their transcription, but with lower transcription efficiency.

Avermectin production in Dsig8 was very close to that in *sig8sav_742* double deletion mutant Dsig8–742 (Supplementary Figure S3), but was higher than that in *sav_742* deletion mutant D742 (Sun et al., 2016), suggesting that other σ^8^ targets may also affect avermectin production in Dsig8.

### σ^8^ Responds to Various Environmental Stresses

**FIGURE 3:**
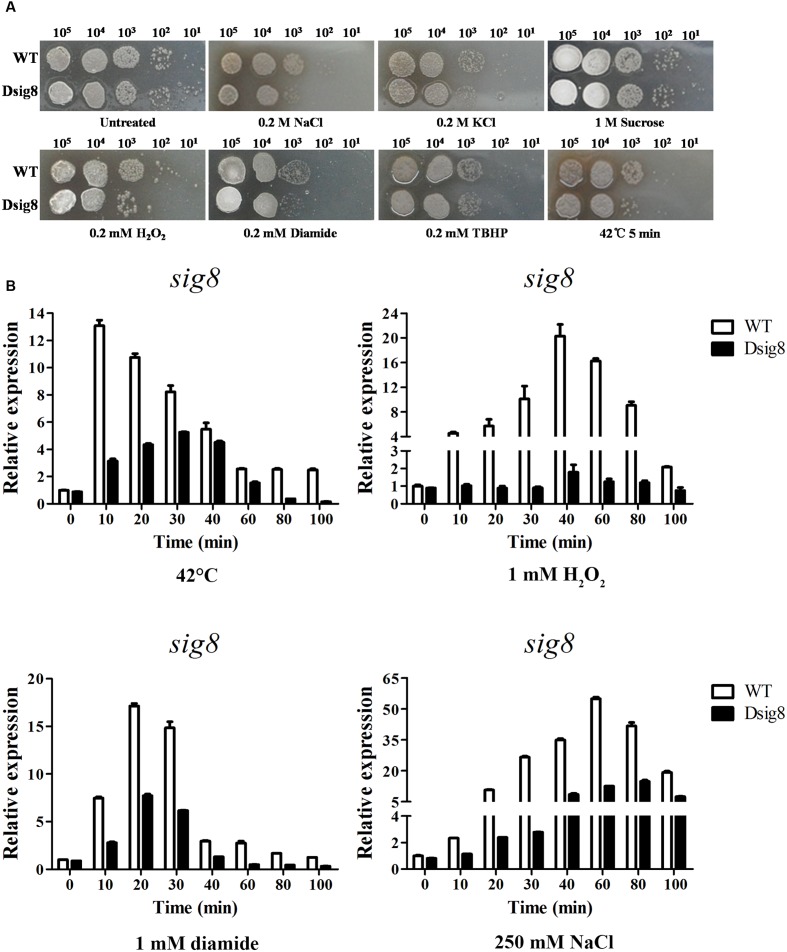
**Role of σ^8^ in responses to osmotic, oxidative and heat stresses.** **(A)** Sensitivity of WT and Dsig8 to stress conditions. For osmotic and oxidative stress assays, serial dilutions of spores were spotted onto YMS plates containing NaCl (0.2 M), KCl (0.2 M), sucrose (1 M), H_2_O_2_ (0.2 mM), diamide (0.2 mM), or TBHP (0.2 mM), and incubated at 28°C for 3 days. For heat stress assay, spore suspensions were treated at 42°C for 5 min and then spotted onto YMS plates. Each stress assay was repeated three times on YMS plates with consistent results. **(B)** Induction of *sig8* transcription in WT and Dsig8 by stresses. RNAs used for qRT-PCR analysis were prepared from cells grown in FM-II for 2 days followed by treatment with 42°C, 1 mM H_2_O_2_, 1 mM diamide, or 250 mM NaCl for the indicated times. Relative values are shown as fold change relative to *sig8* transcription level at the first time point (0 min) in WT, which was assigned as 1.

Alternative σ factors are usually involved in modulation of stress responses (Osterberg et al., 2011). To determine which type(s) of stress σ^8^ responds to, we performed stress tests on YMS plates. Relative to growth of WT, that of *sig8* deletion mutant Dsig8 was more sensitive to NaCl, KCl, H_2_O_2_, diamide, and heat (42°C) stresses, but similarly sensitive to *tert*-butyl hydroperoxide (TBHP) and sucrose stresses (**Figure 3A**). These findings indicate that σ^8^ is required for WT levels of resistance to multiple stresses, e.g., heat stress, salt stress, and oxidative stress (from H_2_O_2_ or diamide).

The observations that *sig8* mutant Dsig8 was more sensitive to various environmental stresses, and that σ^8^ was autoregulated, suggested that *sig8* itself may be induced by these environmental stresses in a σ^8^-dependent manner. We tested this possibility by qRT-PCR comparison of *sig8* transcription levels in WT and Dsig8 under stress conditions. Cells were cultured in soluble FM-II for 2 days, and then treated with heat (42°C), H_2_O_2_, diamide, or NaCl. RNA samples were isolated from cells before (0 min) and after treatment (10, 20, 30, 40, 60, 80, and 100 min). For WT, *sig8* transcription level increased to a maximal value within 10–60 min for each stress type (∼13-fold for heat; ∼20-fold for H_2_O_2_, ∼17-fold for diamide; ∼54-fold for NaCl). For Dsig8, maximal induction was sharply decreased under each stress type (∼6-fold for heat; ∼2-fold for H_2_O_2_, ∼8-fold for diamide; ∼17-fold for NaCl) and delayed for heat (from 10 to 30 min) and salt (from 60 to 80 min) (**Figure 3B**). These findings indicate that σ^8^ itself responds to various environmental stresses at the transcription level in either a σ^8^-dependent or σ^8^-independent manner. The rapid and robust induction of *sig8* in WT may be due to σ^8^-dependent control, whereas the decreased and/or delayed induction in Dsig8 may be due to σ^8^-independent control.

### Identification of σ^8^ Target Genes Related to Heat Stress

**FIGURE 4:**
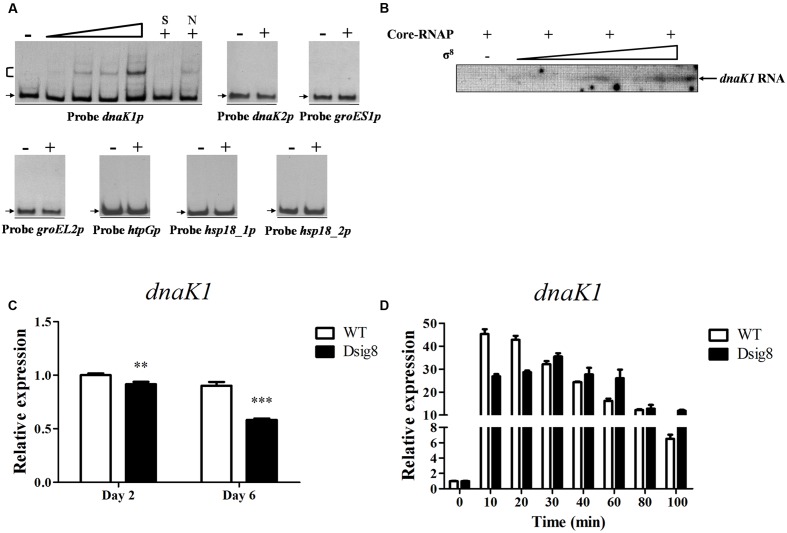
**σ^8^-dependent transcription of *dnaK1*.** **(A)** EMSAs of His_6_-σ^8^ with *dnaK1*, *dnaK2*, *groES1*, *groEL2*, *htpG*, *hsp18_1*, and *hsp18_2* promoter regions under conditions as described for **Figure 2A**. **(B)** *In vitro* transcription assay of the *dnaK1* promoter by Eσ^8^. **(C)** qRT-PCR analysis of *dnaK1* in WT and Dsig8 grown in FM-I. The WT value at day 2 was defined as 1. ^∗∗^, *P* < 0.01; ^∗∗∗^, *P* < 0.001 (*t-*test). **(D)** Induction of *dnaK1* by heat (42°C) in WT and Dsig8 grown in FM-II. *dnaK1* transcription level before temperature rise (0 min) in WT was assigned as 1.

σ^8^ was strongly induced in response to a variety of stresses, suggesting that it mediates defensive systems against stresses. To identify σ^8^ target genes that respond to heat stress, we initially performed a series of EMSAs using His_6_-σ^8^ and potential promoter probes of heat shock genes, including *dnaK1p* for *dnaK1-grpE1-dnaJ1-hspR* operon, *dnaK2p* for *dnaK2-grpE2-dnaJ2* operon, *groES1p* for *groES1-groEL1* operon, *groEL2p*, *htpGp*, *hsp18_1p* and *hsp18_2p*. His_6_-σ^8^ bound specifically to *dnaK1p*, but not to other probes tested (**Figure 4A**). *In vitro* transcription analysis confirmed that *dnaK1* transcription was initiated by σ^8^ (**Figure 4B**). *dnaK1* transcription level in Dsig8 was downregulated at two time points (**Figure 4C**). These findings demonstrate that σ^8^ is a direct activator of *dnaK1*.

*grpE1, dnaJ1*, and *hspR* are cotranscribed with *dnaK1*, and therefore are also σ^8^ targets. *dnaK1*, *grpE1*, and *dnaJ1* encode molecular chaperones for interaction with denatured proteins and facilitate refolding to the native state following heat stress, and *hspR* encodes a putative transcriptional repressor of its operon. Transcription levels of *dnaK1* in WT and Dsig8 under heat stress were recorded to determine whether *dnaK1p* for its operon was induced in a σ^8^-dependent manner. In WT, *dnaK1* transcription level increased rapidly to ∼45-fold after 10 min exposure to 42°C, and then gradually declined. In Dsig8, maximal induction was reduced to ∼35-fold and delayed after 30 min (**Figure 4D**). These findings indicate that σ^8^ facilitates rapid adaption to heat stress mainly by direct regulation of the *dnaK1-grpE1-dnaJ1-hspR* operon. The slower, lower-degree induction of *dnaK1p* in Dsig8 presumably depends on other factors not addressed here.

### Identification of σ^8^-dependent Genes Involved in Responses to Oxidative Stress

**FIGURE 5:**
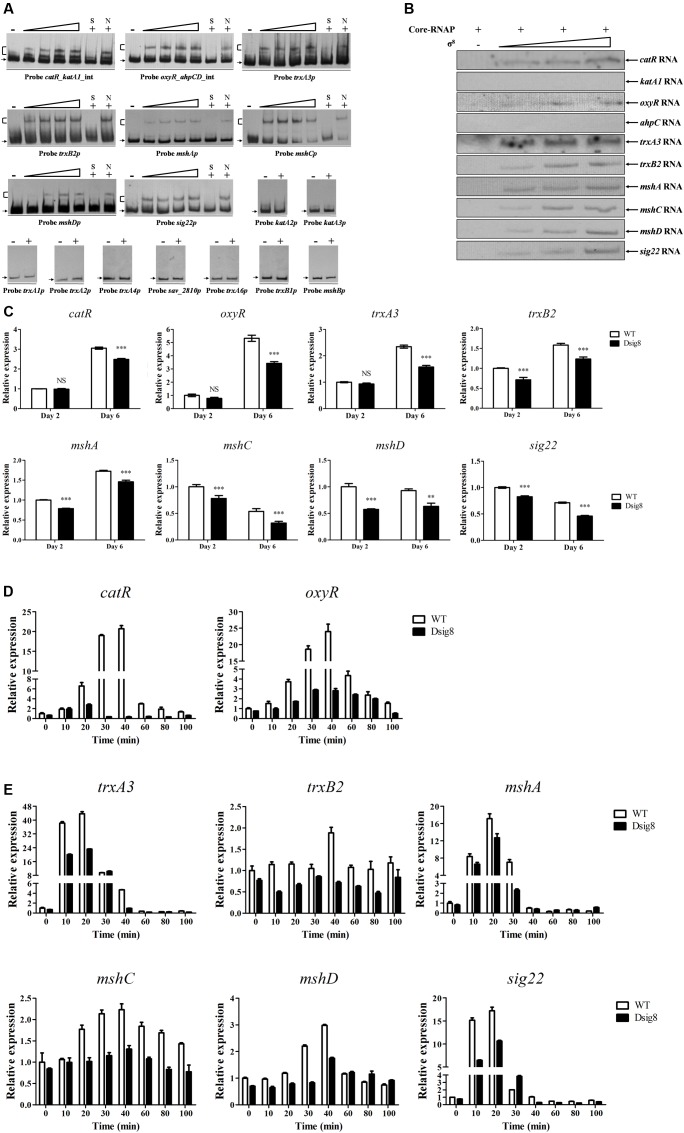
**Identification of σ^8^ target genes involved in responses to oxidative stress.** **(A)** EMSAs of His_6_-σ^8^ with promoters of genes involved in oxidative stress responses. **(B)** *In vitro* transcription analysis of *catR*, *katA1*, *oxyR*, *ahpC*, *trxA3*, *trxB2*, *mshA*, *mshC*, *mshD*, and *sig22* promoters by Eσ^8^. **(C)** qRT-PCR analysis of eight newly identified σ^8^ target genes in WT and Dsig8 grown in FM-I. Transcription level of each gene is expressed relative to WT value at day 2, defined as 1. NS, not significant; ^∗∗^, *P* < 0.01; ^∗∗∗^, *P* < 0.001 (*t-*test). **(D)** Induction of *catR* and *oxyR* by 1 mM H_2_O_2_ in WT and Dsig8 grown in FM-II. Relative value of each gene in WT before treatment (0 min) was assigned as 1. **(E)** Induction of *trxA3*, *trxB2*, *mshA*, *mshC*, *mshD*, and *sig22* by 1 mM diamide in WT and Dsig8 grown in FM-II.

σ^8^ responds to H_2_O_2_ and diamide, which respectively cause peroxidative and thiol-oxidative stress. Bacteria often respond to H_2_O_2_ stress by producing catalases and peroxidases that degrade H_2_O_2_. We showed that responses to H_2_O_2_ stress in *S. avermitilis* involve three catalase genes (*katA1, katA2*, *katA3*), the *ahpCD* operon (encoding alkyl hydroperoxide reductase and alkylhydroperoxidase), and two peroxide-sensing transcriptional factor genes (*oxyR*, *catR*) (Liu et al., 2016). In *S. coelicolor*, ECF-σ^R^ plays a key role in response to thiol-oxidative stress, and major σ^R^ targets include *trx* genes (encoding the thioredoxin system) and *msh* genes (for biosynthesis of mycothiol, the major thiol buffer) (Kallifidas et al., 2010). *S. avermitilis* contains one *sigR* homologous gene (*sig22*), six thioredoxin genes (*trxA1*, *trxA2*, *trxA3*, *trxA4*, *trxA5*, *trxA6*), two thioredoxin reductase genes (*trxB1*, *trxB2*), and four *msh* genes (*mshA*, *mshB*, *mshC*, *mshD*). Possible interactions of σ^8^ with these oxidative stress-related genes were investigated by EMSAs. His_6_-σ^8^ bound specifically to the bidirectional promoter probes *catR_katA1_*int and *oxyR_ahpCD*_int, and to promoter probes *trxA3p*, *trxB2p*, *mshAp*, *mshCp*, *mshDp* and *sig22p*, but not to probes *katA2p*, *katA3p*, *trxA1p*, *trxA2p*, *trxA4p*, *sav_2810p* for *sav_2810-2809-trxA5-2807-cyp12* operon, *trxA6p*, *trxB1p*, or *mshBp* (**Figure 5A**). *In vitro* transcription analysis showed that σ^8^ initiated transcription of *catR, oxyR, trxA3*, *trxB2*, *mshA*, *mshC*, *mshD* and *sig22*, but not that of *katA1* or *ahpC* (**Figure 5B**). Transcription levels of *catR, oxyR, trxA3*, *trxB2*, *mshA*, *mshC*, *mshD*, and *sig22* were all reduced in Dsig8 relative to WT on day 6, or days 2 and 6 (**Figure 5C**). These findings indicate that *catR, oxyR, trxA3*, *trxB2*, *mshA*, *mshC*, *mshD*, and *sig22* are directly activated by σ^8^.

For analysis of oxidative stress responses, WT and Dsig8 were treated with 1 mM H_2_O_2_ or diamide for various durations. In WT, *catR* and *oxyR* were induced to maximal transcription level (∼20- and ∼23-fold, respectively) by H_2_O_2_ within 40 min (**Figure 5D**). In Dsig8, H_2_O_2_ treatment caused only a slight increase of transcription level (∼4-fold induction) of these two genes. These findings indicate that σ^8^ mediates H_2_O_2_ induction of the peroxide-sensing regulators CatR and OxyR. Diamide treatment of WT caused notable induction of *trxA3* (∼43-fold), *mshA* (∼17-fold) and *sig22* (∼17-fold) within 20 min, and slight induction of *mshC* (∼2.2-fold), *mshD* (∼3-fold) and *trxB2* (∼1.8-fold) within 40 min, whereas diamide treatment of Dsig8 had much lower (for *trxA3*, *mshA*, *mshC*, *mshD*, and *sig22*) or no effect (for *trxB2*) on expression of these genes (**Figure 5E**). These findings indicate that σ^8^ helps *S. avermitilis* to cope with thiol-oxidative stress by activating transcription of its target genes *trxA3*, *trxB2*, *mshA*, *mshC*, *mshD*, and *sig22.*

### Identification of σ^8^-dependent Genes Involved in Responses to Osmotic Stress

**FIGURE 6:**
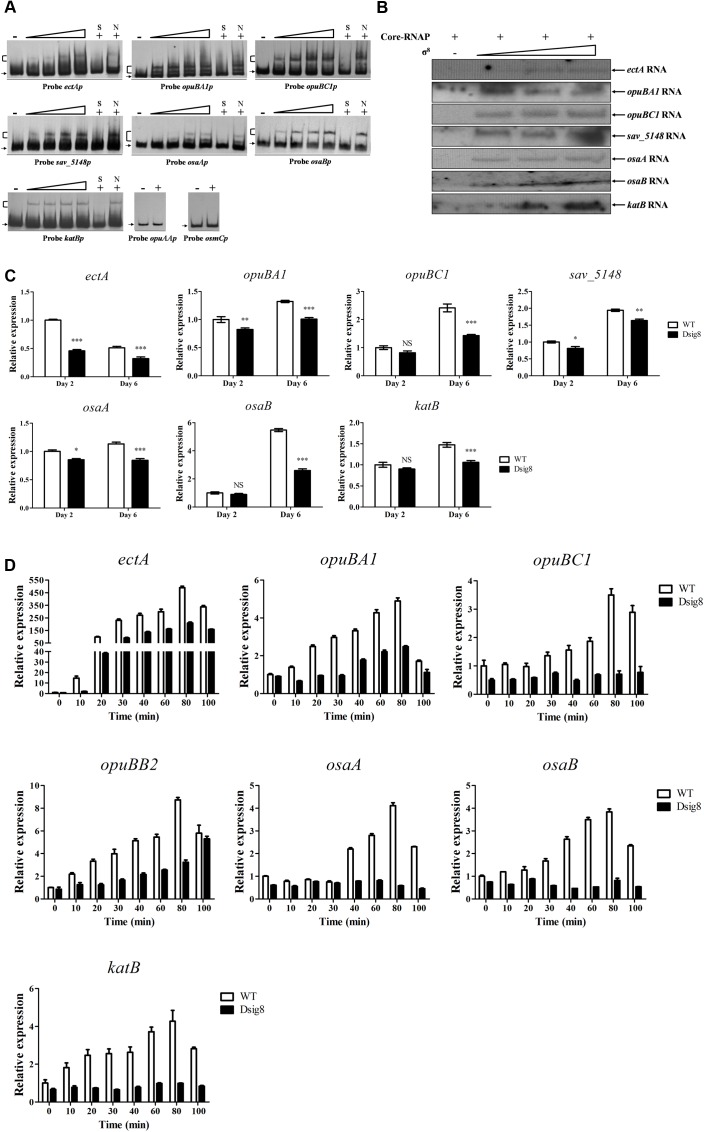
**Identification of σ^8^ target genes involved in osmoprotection.** **(A)** EMSAs of His_6_-σ^8^ with promoters of genes involved in osmoprotection. **(B)** *In vitro* transcription analysis of *ectA*, *opuBA1*, *opuBC1*, *sav_5148*, *osaA*, *osaB*, and *katB* promoters by Eσ^8^. **(C)** qRT-PCR analysis of *ectA*, *opuBA1*, *opuBC1*, *opuBB2*, *osaA*, *osaB*, and *katB* in WT and Dsig8 grown in FM-I. Transcription level of each gene is expressed relative to WT value at day 2, defined as 1. NS, not significant; ^∗^, *P* < 0.05; ^∗∗^, *P* < 0.01; ^∗∗∗^, *P* < 0.001 (*t-*test). **(D)** Induction of seven newly identified σ^8^ target genes by 250 mM NaCl in WT and Dsig8 grown in FM-II. Relative value of each gene in WT before NaCl treatment (0 min) was assigned as 1.

The σ^8^ homolog, *S. coelicolor* σ^B^, responds to osmotic stress caused by NaCl and KCl. To investigate σ^8^ target genes involved in osmoprotective functions, a series of candidates were selected for EMSA analysis: *ect* genes (encoding enzymes for biosynthesis of ectoine) (Bursy et al., 2008), *opu* genes (encoding ABC transporters for uptake of osmoprotectants) (Horn et al., 2005), *osmC* (encoding a putative osmotically inducible protein) (Gutierrez and Devedjian, 1991), *osaAB* (encoding a two-component system) and *katB* (encoding a catalase). *osaAB* and *catB* (*katB* homolog) were reported to be essential for osmoadaptation in *S. coelicolor* (Cho et al., 2000; Bishop et al., 2004). EMSAs revealed specific binding of His_6_-σ^8^ to the promoter probes *ectAp* for *ectA-ectB-ectC-ectD* operon, *opuBA1p*, *opuBC1p*, *sav_5148p* for *sav_5148-opuBB2-opuBA2-opuBB1-opuBC2-sav_5143* operon, *osaAp*, *osaBp*, and *katBp*, but not to those of *opuAAp* for *opuAA-opuAB* operon or *osmCp* (**Figure 6A**). *In vitro* transcription assay confirmed initiation of transcription of *ectA*, *opuBA1*, *opuBC1*, *sav_5148*, *osaA*, *osaB*, and *katB* by σ^8^ (**Figure 6B**). Transcription levels of seven newly identified σ^8^ target genes were all lower in Dsig8 than in WT (**Figure 6C**), demonstrating the role of σ^8^ as an activator of these genes. *ectB, ectC*, and *ectD* are cotranscribed with *ectA*, and *opuBB2*, *opuBA2*, *opuBB1*, and *opuBC2* are cotranscribed with *sav_5148*; therefore, these are also target genes of σ^8^.

σ^8^ may also affect induction of its target osmoprotection-related genes by salt stress in *S. avermitilis. ectA* transcription in WT was sharply increased to ∼490-fold by 80 min of NaCl treatment (**Figure 6D**), suggesting that ectoine plays as a key osmoprotective role. Maximal induction by NaCl treatment in WT was ∼3.5-fold to 8.7-fold for *opuBA1*, *opuBC1*, *opuBB2* (selected for qRT-PCR analysis for its operon), *osaA*, *osaB*, and *katB* (**Figure 6D**). In Dsig8, NaCl treatment had no effect on transcription of *opuBC1*, *osaA*, *osaB*, or *katB*, indicating that salt stress induces these genes in a σ^8^-dependent manner. Maximal induction by NaCl treatment on *ectA*, *opuBA1*, and *opuBB2* transcription was lower in Dsig8 than in WT, suggesting that these three genes are induced by salt stress in both σ^8^-dependent and σ^8^-independent manners.

### Prediction of the σ^8^ Regulon

**FIGURE 7:**
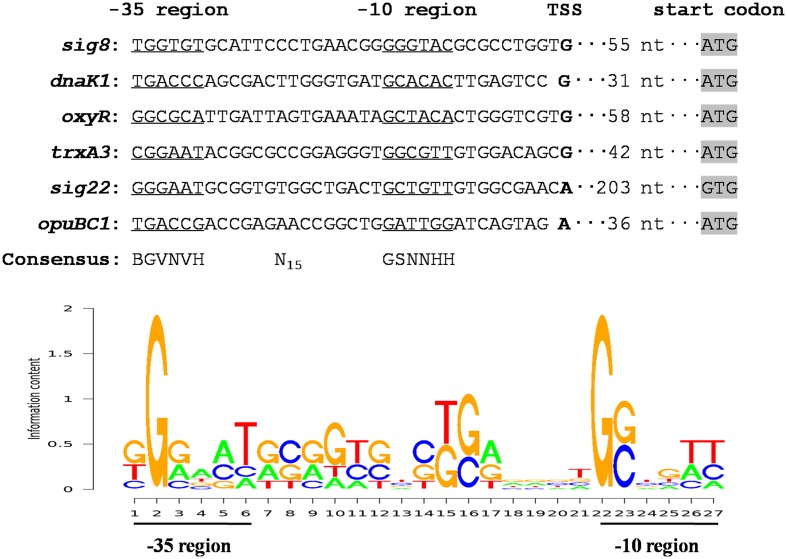
**Analysis of consensus σ^8^-binding promoter sequence using the PREDetector program.** Putative -35 and -10 regions are underlined. Shading: translational start codons. Boldface: TSSs. In the sequence logo of σ^8^-binding consensus, appearance frequency of each base is proportional to the height of the corresponding letter.

σ^8^ in *S. avermitilis* evidently plays a pleiotropic role in control of avermectin production and in protection against a variety of stresses. To clarify broader roles of σ^8^ in this species, more σ^8^ target genes need to be identified. The recognition and binding sites of σ^70^-family factors are the -35 and -10 hexamers of its target promoters. We conducted 5′-RACE assays to determine promoter structures of several σ^8^ targets and identified a consensus σ^8^-binding sequence. TSSs of *sig8*, *dnaK1*, *oxyR*, *trxA3*, *sig22*, and *opuBC1* were determined by 5′-RACE analysis of WT gene transcripts under stress conditions (Supplementary Figure S4), leading to the putative -35 and -10 promoter sequences shown in **Figure 7**. Analysis of these promoter sequences using the PREDetector web-based program^1^ revealed a consensus sequence BGVNVH-N_15_-GSNNHH (**Figure 7**), which resembles that of σ^B^-specific promoters of *S. coelicolor* (GNNTN-N_14-16_-GGGTAY) (Y: C or T) (Lee et al., 2004b).

RNA polymerase is not sensitive to variations in spacer length between the -35 and -10 regions, and the consensus σ^B^-binding sequence is GNNTN-N_14-16_-GGGTAY. We therefore employed the sequence pattern BGVNVH-N_14-16_-GSNNHH to scan the *S. avermitilis* genome using the PREDetector program to predict candidate members of the σ^8^ regulon, and selected putative promoters that were located within 300 nt upstream of the translational start codons. Using a cut-off score of ≥6.5, we identified 940 putative σ^8^ target genes, of which 453 have unknown function or are unclassified (Supplementary Table S2). The remaining 487 genes were assigned to 17 functional groups. Among these 487 genes, 179 are associated with regulatory functions, according to the KEGG pathways database for *S. avermitilis*^2^. These findings suggest the extent biological significance of σ^8^ in *S. avermitilis*.

## Discussion

Important roles of alternative σ factors in *Streptomyces* species in regulation of secondary metabolism are suggested by several previous studies. In *S. coelicolor*, σ^B^ (Cho et al., 2001; Lee et al., 2004b) and σ^K^ (Mao et al., 2009) are involved in regulation of ACT and RED biosynthesis, although the regulatory mechanisms are unclear. In *Streptomyces chattanoogensis* L10, alternative σ factor WhiG_ch_ promotes natamycin biosynthesis by directly activating two structural genes, *scnC* and *scnD* (Liu S. P. et al., 2015). In the present study, we characterized alternative σ factor σ^8^ in *S. avermitilis*, and demonstrated that it indirectly represses avermectin production through its effects on expression of CSR gene *aveR* and structural gene *aveA1*. σ^8^ is the first among the 10 alternative σ factors in *S. avermitilis* to be characterized. Our findings augment the limited knowledge of regulation of secondary metabolism by alternative σ factors in *Streptomyces*.

AveR is an essential activator for transcription of the *ave* cluster (Guo et al., 2010). We showed that σ^8^ initiates transcription of SAV742, which directly represses expression of *ave* structural genes *aveA1*, *aveD*, *aveF*, and *aveA4* (Sun et al., 2016), but not that of *aveR*. Thus, σ^8^ controls avermectin production through at least two pathways: (i) directly initiating transcription of *sav_742*; (ii) indirectly repressing expression of *aveR*. The observation that σ^8^ has an indirect effect on *aveR* expression suggests that it regulates *aveR* through a “cascade” mechanism. However, our search for *aveR* upstream regulator(s) that mediate the repression of σ^8^ on *aveR* showed that the two known *aveR* direct repressor genes, *phoP* (Yang et al., 2015) and *avaR2* (Zhu et al., 2016), are not targets of σ^8^. Future studies will identify such direct regulator(s) of *aveR* and clarify the mechanisms underlying σ^8^ function in avermectin production.

Alternative σ factors help to control morphological differentiation and stress responses, in addition to secondary metabolism. In *S. coelicolor*, σ^B^ is required for aerial mycelium formation and osmoprotection (Cho et al., 2001); σ^B^-dependent σ^L^ and σ^M^ are involved in sporulation; σ^H^ is activated under heat stress and osmotic stress, and plays a crucial role in morphological development (Sevcikova et al., 2001); σ^I^ responds to osmotic stress, but has no effect on differentiation (Homerova et al., 2012); σ^F^ and σ^WhiG^ affect spore formation (Chater et al., 1989; Potuckova et al., 1995; Lee et al., 2005). In *S. hygroscopicus*, *sigB* is induced by heat stress and ROS inhibitor (Wei et al., 2012). The present study showed that σ^8^ is involved in protection against heat, osmotic and oxidative stresses, but has no effect on morphological differentiation. The differing functions of σ^B^ homologes in various *Streptomyces* species presumably reflect differences in regulatory mechanisms for σ^B^ activity/expression, and in σ^B^-mediated regulatory pathways for adaptation to various environmental stresses.

In many Gram-positive bacteria, activity of σ^B^ homologs is modulated by their cognate anti-σ factors (Hecker et al., 2007), which inhibit transcription activity of σ^B^ by binding to it. Under stress conditions, σ^B^ is released free of its anti-σ factor, and subsequently initiates transcription of its target genes related to stress protection. The anti-σ factor gene is typically located adjacent to the *sigB* locus. In *S. coelicolor*, the anti-σ^B^ factor gene *rsbA* (*sco0599*) is upstream of *sigB* (*sco0600*) (Lee et al., 2004a). In *S. avermitilis*, the *rsbA*-homolog gene *prsR* (*sav_7158*) is not located near *sig8* (*sav_741*), and *sig8*-adjacent genes have no similarity to *rsbA*, suggesting that the regulatory mechanism of σ^8^ activity is different from that of σ^B^. Further studies to identify regulators associated with σ^8^, based on co-immunoprecipitation assays within *S. avermitilis* cells, are in progress.

σ^8^ responds rapidly to heat stress by directly activating transcription of the *dnaK1-grpE1-dnaJ1-hspR* operon. Heat shock proteins include not only chaperones for refolding denatured proteins, but also proteases for degrading more denatured proteins. *S. avermitilis* has three putative heat shock protease genes *lonA*, *htpX1*, and *htpX2*, and whether they are under direct control of σ^8^ needs to be investigated. Under H_2_O_2_ stress, σ^8^ directly activates transcription of H_2_O_2_-sensing regulator genes *catR* and *oxyR*, but not of *kat* or *ahpCD*, suggesting that CatR and OxyR mediate H_2_O_2_ induction of peroxide-scavenging enzymes. We showed that OxyR in *S. avermitilis* directly activates expression of antioxidant enzyme genes *ahpCD*, *katA1*, *katA2*, and *katA3* in response to H_2_O_2_ stress (Liu et al., 2016). The targets of CatR remain to be characterized. σ^8^ thus exerts its protective effect against H_2_O_2_ damage in *S. avermitilis* mainly through a cascade regulatory mechanism involving control of OxyR. σ^R^ homologes and their predicted targets, e.g., *trx* and *msh* genes are well-conserved in *Streptomyces* (Kim et al., 2012). Two *trx* genes, three *msh* genes and *sig22* (*sigR* homolog) in *S. avermitilis* are directly controlled by σ^8^ in response to diamide, indicating that σ^8^ facilitates a rapid response to thiol-oxidative stress through both direct and cascade regulatory mechanisms. σ^8^ targets involved in osmoprotection, identified in this study, are *ect* genes, *opuB* genes, *osaAB* and *katB*. The specific substrate of OpuB transporter in *S. avermitilis* remains unknown. Our findings are consistent with the observations that ectoine is osmoprotectant, and *osaAB* and *katB* are required for σ^B^-dependent osmoprotection in *S. coelicolor* (Cho et al., 2000, 2001; Bursy et al., 2008; Fernández Martínez et al., 2009), indicating a conserved role of ectoine, *osaAB* and *katB* in osmoadaptation in *Streptomyces.*

**FIGURE 8:**
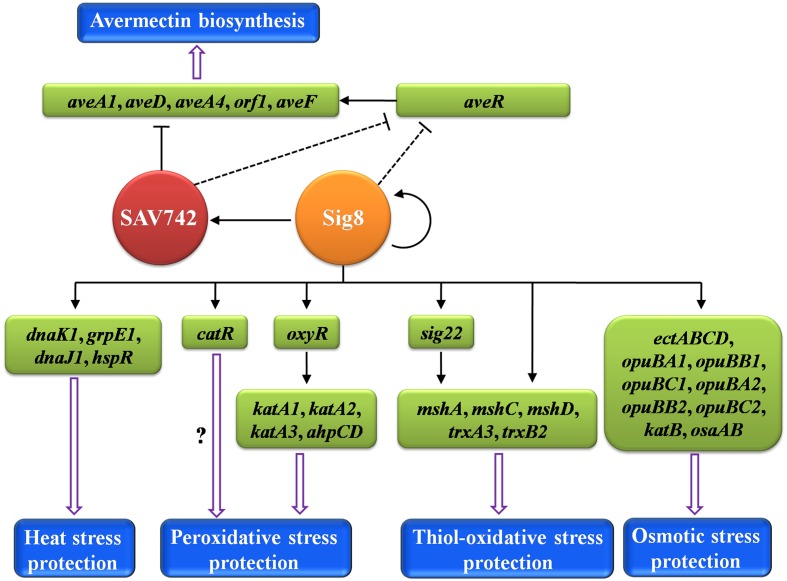
**Proposed model of σ^8^-mediated regulatory network in *S. avermitilis*.** σ^8^ exerts pleiotropic regulatory effects on avermectin biosynthesis and responses to four types of stress. Solid arrows: direct activation. Solid bars: direct repression. Dashed bars: indirect repression. Hollow arrows: avermectin biosynthesis or response to stress.

Our present findings, taken together, demonstrate clearly that σ^8^ is a pleiotropic regulator of avermectin production and responses to heat, osmotic and oxidative stresses in *S. avermitilis*. A proposed model of the σ^8^-mediated regulatory network is presented in **Figure 8**. σ^8^ is a good example of a regulator that links stress responses to antibiotic production. Regulatory pathways of specific stress responses are potential targets for genetic manipulation to increase antibiotic yields. For example, disruption of *osaB* led to a 37% increase in avermectin yield (Godinez et al., 2015), and deletion of *sig8* increased avermectin yield ∼96%. Continued elucidation of such regulatory mechanisms will contribute to improvements in antibiotic production.

Using the identified σ^8^ consensus binding sequence, we predicted 940 putative σ^8^ target genes. It is unlikely that σ^8^ binds to all the predicted targets, more knowledge of the promoter sequence recognized by σ^8^ is necessary for precise prediction of σ^8^ regulon. Studies using high-throughput technologies (e.g., ChIP-seq) are in progress to experimentally verify additional σ^8^ targets in *S. avermitilis* and thereby elucidate the wide range of cellular functions controlled by this important σ factor.

## Author Contributions

YW and DS: designed the research, DS: performed experiments, QW, ZC, and JL: contributed study materials, YW and DS: wrote the manuscript.

## Conflict of Interest Statement

The authors declare that the research was conducted in the absence of any commercial or financial relationships that could be construed as a potential conflict of interest.

## Footnotes

1 http://predetector.hedera22.com/

2 www.genome.jp/kegg-bin/show_organism?org=sma
