# Supplementary material for: An Alternative σ Factor, σ8, Controls Avermectin Production and Multiple Stress Responses in Streptomyces avermitilis
Source: Front Microbiol. 2017 Apr 24;8:736. doi: 10.3389/fmicb.2017.00736 (PMC5402319; doi:10.3389/fmicb.2017.00736)
Supplement: Supplementary file 1 [file Presentation_1.PDF]

Supplemental Materials

An Alternative σ Factor, σ^8, Controls Avermectin Production and Multiple Stress Responses in *Streptomyces avermitilis*

Di Sun, Qian Wang, Zhi Chen, Jilun Li and Ying Wen*

State Key Laboratory of Agrobiotechnology and MOA Key Laboratory of Soil Microbiology, College of Biological Sciences, China Agricultural University, Beijing, China

*** Correspondence:**

Ying Wen

E-mail: wen@cau.edu.cn   Phone: +86-10-62732715

Supplementary Figures:

FIGURE S1: Method (schematic) for *sig8* deletion.

FIGURE S2: Phenotypes of WT, Dsig8, Csig8 and Osig8 grown on YMS, MM, or R2YE plates at 28°C.

FIGURE S3: Comparative avermectin yield in WT, Dsig8, D742 and Dsig8-742 grown in FM-I for 10 days.

FIGURE S4: Determination of TSSs of *sig8*, *dnaK1*, *oxyR*, *trxA3*, *sig22* and *opuBC1* by 5′-RACE PCR.

Supplementary Tables:

TABLE S1: Primers used in this study.

TABLE S2: Putative targets of σ^8.

FIGURE S1

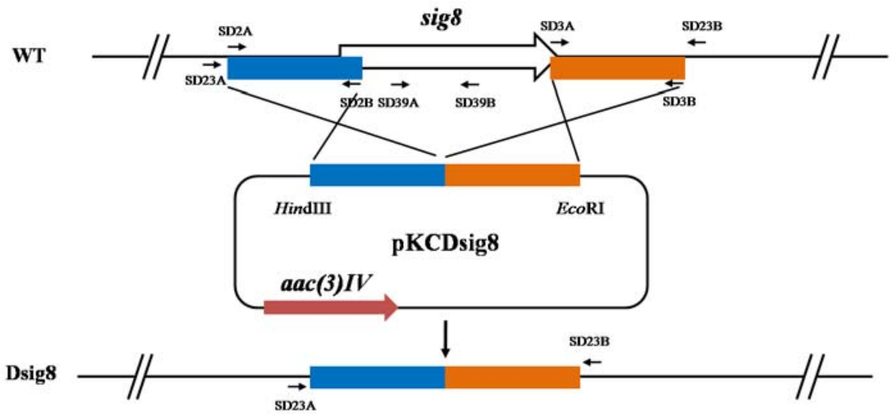

FIGURE S1 | Method (schematic) for *sig8* deletion. Long arrows: genes and their transcription directions. Short arrows: primer positions. Blocks: homologous exchange regions used for *sig8* deletion.

FIGURE S2

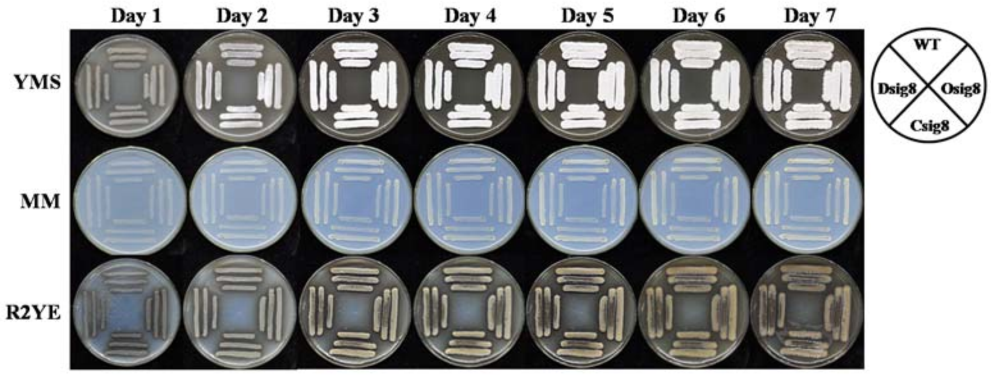

FIGURE S2 | Phenotypes of WT, Dsig8, Csig8 and Osig8 grown on YMS, MM, or R2YE plates at 28°C.

FIGURE S3

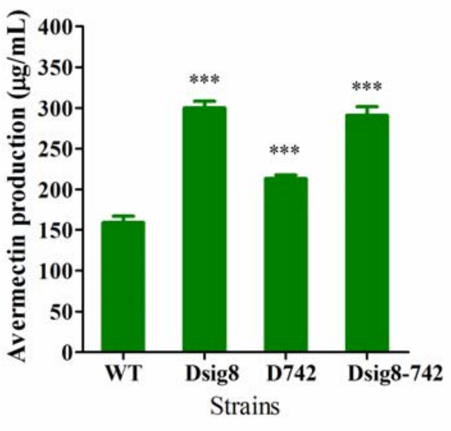

FIGURE S3 | Comparative avermectin yield in WT, Dsig8, D742 and Dsig8-742 grown in FM-I for 10 days. WT: wild-type strain ATCC31267. Dsig8: *sig8* deletion mutant. D742: *sav_742* deletion mutant. Dsig8-742: *sig8sav_742* double deletion mutant. Error bar: SD from three independent experiments. ***, $P < 0.001$ (Student's *t*-test).

FIGURE S4

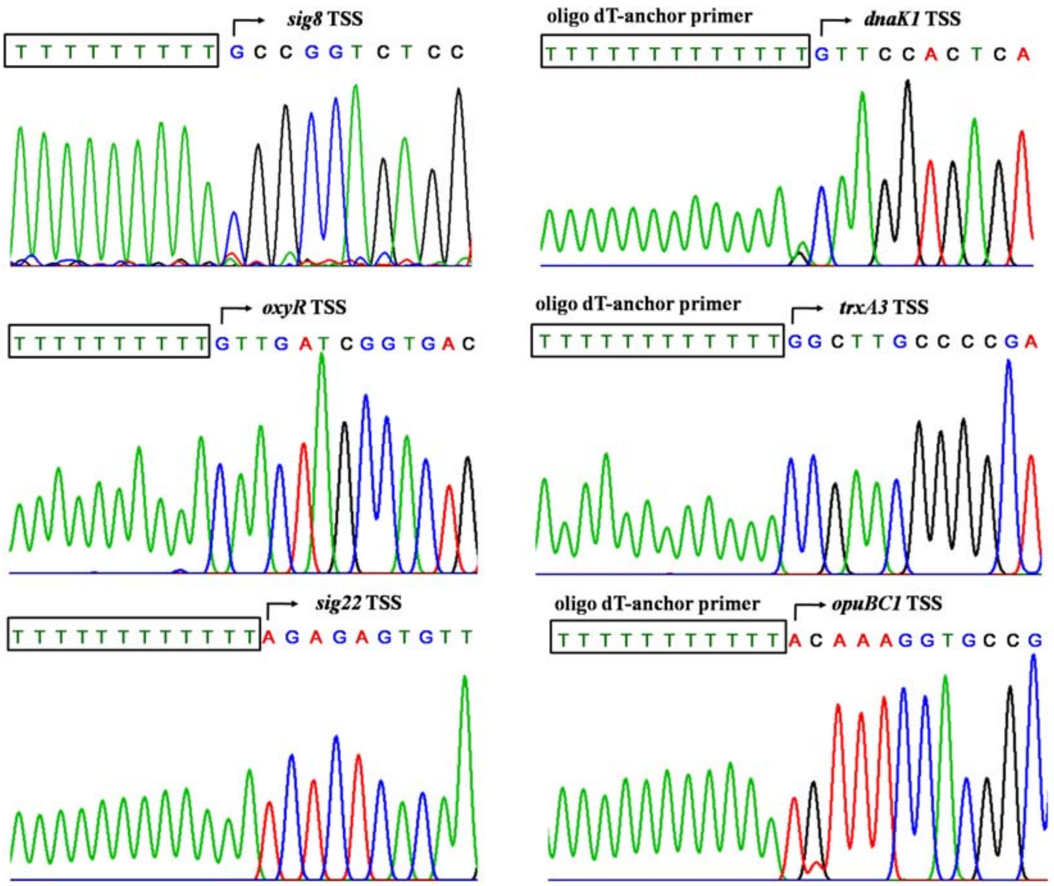

FIGURE S4 | Determination of TSSs of *sig8*, *dnaK1*, *oxyR*, *trxA3*, *sig22* and *opuBC1* by 5′-RACE PCR. Boxed areas: sequences of 5′-RACE oligo dT-anchor primers. Bent arrows: bases of TSSs. RNA samples were isolated from WT cells grown in FM-II for 2 days, followed by treatment with heat (42°C) for 10 min (for analysis of *dnaK1* TSS), H_2O_2 for 40 min (for analysis of *oxyR* and *sig8* TSSs), diamide for 20 min (for analysis of *trxA3* and *sig22* TSSs), or NaCl for 80 min (for analysis of *opuBC1* TSS), to ensure that tested genes were robustly transcribed.

TABLE S1 | Primers used in this study.

Primer purpose/primer	DNA sequence* (5'-3')	Use
Gene disruption, complementation and overexpression		
SD2A	CCCAAGCTTATGTCGACCTCACGAAGGAG (*Hin*dIII)	Deletion of *sig8* gene
SD2B	CGCGGATCCGACGAGGGTGACGTTCATCT (*Bam*HI)	
SD3A	CGCGGATCCAACTGACCCCACTCGTTCG (*Bam*HI)	
SD3B	CCGGAATTCCCTAACTCCATCAGCCGGTA (*Eco*RI)	
SD23A	GGGAAAGTCGACAAGCACTC	Confirmation of *sig8*
SD23B	GGATCGGACTGACGTACCAT	deletion in Dsig8
SD39A	CCTATATCGTCGGTGAGATCAAG	
SD39B	CGAACCGCATGTGGATGATG	
SD1D	TGCGGATCCCTACTACCAGAGCCACCGAC (*Bam*HI)	Complementation of
SD1B	CCCAAGCTTGGCCGAGCGATTTTGCGTT (*Hin*dIII)	Dsig8
SD1C	TGCGGATCCCACGGGGGCTGGATACAC (*Bam*HI)	Overexpression of *sig8*
SD1B	CCCAAGCTTGGCCGAGCGATTTTGCGTT (*Hin*dIII)	in *S. avermitilis*
SD1A	CGGGATCCATGACAGTGACCGAGGCAACA (*Bam*HI)	Overexpression of
SD1B	CCCAAGCTTGGCCGAGCGATTTTGCGTT (*Hin*dIII)	His_6-σ^8 protein in *E. coli*
5'-RACE		
Oligo-dT anchor primer	GACCACGCGTATCGATGCGCACTTTTTTTTTTTTTTTv	
Anchor primer	GACCACGCGTATCGATGTCGAC	
sig8SP1	CTTCCAGAACGGCGAGCCTG	Identification of TSS of
sig8SP2	TCGGACGGCTGGAGAGTG	*sig8*
sig8SP3	CAGAGCACCGAGGCCCTC	
dnaK1SP1	GATGACCGCGTCCGTGAC	Identification of TSS of
dnaK1SP2	TCATCTGCTGCGGGTTGAAG	*dnaK1*
dnaK1SP3	GAGCACCTCACCGTTCTTGG	
oxyRSP1	GGCGGTCTGTTCCTCGTG	Identification of TSS of
oxyRSP2	AGGACGGTCGGCAGCAGAT	*oxyR*
oxyRSP3	CCCGAGTGTCTCCTCCAGG	
trxA3SP1	CTGTCCGATCACGTCCTC	Identification of TSS of
trxA3SP2	GCGAACACGGCGACCTGG	*trxA3*
trxA3SP3	CGAACACCAGGTCCGGATTG	
sig22SP1	GATCTCCTCGGCCGCGCT	Identification of TSS of
sig22SP2	GGTGCCCTCACGGAACTGG	*sig22*
sig22SP3	CACCTCCTCCAGCGATGT	
opuBC1SP1	GTTGTACTCCGGCAGCAC	Identification of TSS of
opuBC1SP2	GCTTGTAGGTGACCTTGATG	*opuBC1*
opuBC1SP3	GAGCGGGTCGGACTCTTC	
EMSA		
aveRp-Fw	CCGCACCGCCATACATAC	Probe *aveRp*
aveRp-Rev	GAAACTCCCTGCATGATGTTC	

aveA1p-Fw	ATGGTCGGGAACCTCCGCAA	Probe *aveA1p*
aveA1p-Rev	CTGTGTCCTCACCGCTAGGC	
SD74A	CGCAAGATCTATGTCGACCTCAC	Probe *sig8p*
SD74B	GGGGCCGACCAAAGACAC	
SD73A	TGCTCACCACCAACTGACC	Probe *sav_742p*
SD73B	CCCGATCATTCCGTTCATC	
hrdB-Fw	CATCGTTGACCACCTATGACC	Probe *hrdB*
hrdB-Rev	CTCTCGGAACGTTGGAAAAC	
SD215A	GTCCATGCATCCAGGTTAG	Probe *phoRp*
SD215B	GCTCCCAGGTGAACTCTG	
avaR2p-Fw	CAGGGAGGTGCCGGGGG	Probe *avaR2p*
avaR2p-Rev	GCGGAAGGCGGCTGACC	
dnaK1p-Fw	AGGAGCGGATAAGACTTGAGT	Probe *dnaK1p*
dnaK1p-Rev	TTAGTCGTGCCCAGGTCG	
SD335A	CGTTCTCCTCGAGCCTACAGG	Probe *dnaK2p*
SD335B	CTCCCACACGGCGATCAC	
SD339A	ATTGGCACTCCGCTTGAC	Probe *groES1p*
SD339B	GGCTTGATGGCAACCTTG	
SD337A	TTCAGATGGACGGCTACC	Probe *groEL2p*
SD337B	AGAGTGCTAACGCCAATGA	
SD340A	GGAAAGCCGCTGGTCAGAC	Probe *htpGp*
SD340B	GGGAGTCCATCGTCGCAG	
SD341A	ATCGTCCCTGGTCATCGG	Probe *hsp18_1p*
SD341B	ATCAACATCGTGAAACACCTCC	
SD342A	AGTGCTGTCGTCGCTCAT	Probe *hsp18_2p*
SD342B	CATCAACACGGTAAACACCTC	
SD123A	GCGCAGTCGTTCCAGTAG	Probe *catR_katA1*_int
SD123B	GGACTCGCTGTTCTGATTGT	
SD124A	GGAAAGGTGGTCTGGTTC	Probe *katA2p*
SD124B	GGTGTACGGAGCCTTCTG	
SD122A	AGGTCCGTGGAGCGCCTC	Probe *katA3p*
SD122B	CGGTGGCTGAGTTCTGGTTGTC	
SD236A	GAGAAGCCGAGCACCTGG	Probe *oxyR_ahpCD*_int
SD236B	CGCTGGAGAAGGGCAATG	
trxA1p-Fw	CGAACCTGAGCGGTGTCT	Probe *trxA1p*
trxA1p-Rev	AGGAGTCGTCGGTCACAT	
trxA2p-Fw	GCTACATCTCCCACGAGG	Probe *trxA2p*
trxA2p-Rev	TTCGGACAACTGACGGTC	
trxA3p-Fw	GCCGACCAGCTCGCTCTC	Probe *trxA3p*
trxA3p-Rev	GGCGTGTTTCCCTCTTCCTG	
trxA4p-Fw	AATCAAGCATTCCGCACC	Probe *trxA4p*
trxA4p-Rev	TCTCACTCTCCCGCTCCG	
trxA5p-Fw	TCCAGGACTTCGGGTTCG	Probe *sav_2810p*
trxA5p-Rev	GTCACTTCGGTCACGCCC	

trxA6p-Fw	GGGACAGTTACGCTACGAC	Probe *trxA6p*
trxA6p-Rev	TGGAGGCACAGCAGAGAC	
trxB1p-Fw	ATCGCTCTCTTCCCAGGC	Probe *trxB1p*
trxB1p-Rev	GTCGCTCACGGCTTGATT	
trxB2p-Fw	CCAGAAGGGTGTCAGGAG	Probe *trxB2p*
trxB2p-Rew	ACATCAGACGAACGCCTC	
mshAp-Fw	GTTCGGGTTCGTGGTCCC	Probe *mshAp*
mshAp-Rev	GTGGCGTTCCTCCTCGCT	
mshBp-Fw	AGACCTGTGACGGCTGCG	Probe *mshBp*
mshBp-Rev	TGGACCCACCCTACGAGG	
mshCp-Fw	CTCACCTCGGGAAAGCAC	Probe *mshCp*
mshCp-Rev	TAGATACGGGCGACGGGA	
mshDp-Fw	GGAGAGGATGCGGTGGAT	Probe *mshDp*
mshDp-Rev	GCCATACGGTGTCGTCGC	
SD233A	TACGGCGATCATGGTTTC	Probe *sig22p*
SD233B	GATAGACGACGATCCGGTCC	
SD173A	TGACCCCTCCCGCCACTTC	Probe *ectAp*
SD173B	GCGTCGATAACCCCCATTTTGTC	
SD139A	GATTCCACAGCCGTCGAG	Probe *opuBA1p*
SD139B	CGAACTGGATCATGAGGTGTC	
SD138A	AGACTACCGCCTCTTCAGAC	Probe *opuBC1p*
SD138B	GGGTTTCTCGTCCTTGAC	
SD140A	TGGTGTGTGGTCTGCGTC	Probe *sav_5148p*
SD140B	CCAGTGGTCATCCTGTACATG	
SD331A	CGCCAGACTCCACTGTGT	Probe *osaAp*
SD331B	CGGTCACCACTGTTTCGT	
SD141A	TGGAACAGTGGATGCGTG	Probe *osaBp*
SD141B	CAGCGCCAGCAGATTCTC	
SD99A	GAAATGAATCGCCCATGACTTG	Probe *katBp*
SD99B	TTCCGTGCTGCTCGCTTG	
SD333A	GTTGGCTGCGTCACCTTT	Probe *opuAAp*
SD333B	ACCTCTCGTCCTGGAGCG	
SD136A	GAAGAGTTCGACCATGCATG	Probe *osmCp*
SD136B	CACACACCGTCTGGGAAG	
In vitro transcription		
SD121A	TCGGCAGCTACTACCAGAG	*sig8* promoter and
SD121B	GTCAAGCCTGCTTTGGAG	partial ORF
SD157A	GCGAACACCTCGGCGTCT	*sav_742* promoter and
SD157B	CGTCCAGCAGCAGCGTCA	partial ORF
SD80A	CTGACCGGCGGCTACCTG	*aveR* promoter and
SD80B	CTCCCTGCATGATGTTCTTA	partial ORF
SD371A	CTTGCGTGTAAGGAGCGG	*dnaK1* promoter and
SD371B	ACGAGGATGGTCTGGTCG	partial ORF
SD131A	GCGCTCACGGTTGAAGTG	*catR* promoter and

SD131B	TCGCGGAAACGATCAG	partial ORF
SD388A	GCGCTGCGCGGTCATCCGC	*katA1* promoter and
SD388B	GGGATGCGCTCACGGTTG	partial ORF
SD132A	CGTAGGTCTTGTGGTTGAT	*oxyR* promoter and
SD132B	GAAGTCCTCGTCGAACAG	partial ORF
SD365A	CGTATCCCTCACCCATCG	*ahpC* promoter and
SD365B	CGTATCCCTCACCCATCG	partial ORF
SD389A	AGCTCGCTCTCGACGACC	*trxA3* promoter and
SD389B	CGCCGCCTTCTCGTAGAC	partial ORF
SD367A	GAAGGGTGATCCGGATGTC	*trxB2* promoter and
SD367B	CCTCGTCGGTCTCCAGGT	partial ORF
SD368A	CGTTCACGCACCCTTTCG	*mshA* promoter and
SD368B	ATCTCGCCGATGACACGC	partial ORF
SD369A	CTCACCTCGGGAAAGCAC	*mshC* promoter and
SD369B	GGTTCTTCTTGCCAGGACG	partial ORF
SD370A	GAGAGGATGCGGTGGATACGT	*mshD* promoter and
SD370B	CGCTCGGCCAGGAAGAAG	partial ORF
SD366A	CGTGTTGTGGAGTGGACC	*sig22* promoter and
SD366B	AAAGCCCTCTACATCCGC	partial ORF
SD364A	GGCACTTTCCATCGCTTC	*ectA* promoter and
SD364B	CGAACAGGACCTCACGCT	partial ORF
SD155A	GGGCTCACTTCCACGGTC	*opuBA1* promoter and
SD155B	GTTCGTCCTGGAGCTGGG	partial ORF
SD182A	CGTAGATGTCGGCGATCAGGAT	*opuBC1* promoter and
SD182B	GAGCGGGTCGGACTCTTCG	partial ORF
SD181A	ACAAGCTGCATGCCGAAG	*sav_5148* promoter and
SD181B	CATCCGTGAGCAACTGTTG	partial ORF
SD363A	CTTACCTTCGCCGTCCGT	*osaA* promoter and
SD363B	GGTGACCTCGTCGGTGAA	partial ORF
SD390A	TGGAACAGTGGATGCGTGC	*osaB* promoter and
SD390B	CGAAGTCGTCCGTGAGCAGT	partial ORF
SD391A	AAATGAATCGCCCATGACT	*katB* promoter and
SD391B	CTCCGTCGGCTCCGCCAC	partial ORF
qRT-PCR		
16S-QP-Fw	AGCGGAGCATGTGGCTTAAT	*16S rRNA* ORF
16S-QP-Rev	ACGTATTCACCGCAGCAATG	
SD65A	TTGTCCCGGCTGTTCTTC	*sig8* ORF
SD65B	GACGAGGGTGACGTTCATCT	
SD66A	GGGGATCGGGGATGAAC	*sav_742* ORF
SD66B	ACCAATAGTCGAACCGGTTG	
GJ97	CAGAAGAACTCACGCTCGTC	*aveR* ORF
GJ98	ACTCTTTCCACAGCCCATTC	
GJ99	CGGACAGGACTACGCACTTC	*aveA1* ORF
GJ100	ACGAGATACGACCGGAGATC	

SD346A	CCAAGAACGGTGAGGTGC	*dnaK1* ORF
SD346B	CTGCTGCGGGTTGAAGTC	
catR-QP-Fw	TTGAAGCTGCCCGAGATCTCC	*catR* ORF
catR-QP-Rev	CTTGTCCGTGGCGACCTCC	
oxyR-QP-Fw	GATGGAGGAGGCCGAGGC	*oxyR* ORF
oxyR-QP-Rev	GCAGGAGGTTGAGCTCACG	
SD135A	ATGAGCAGCACCGTGGAG	*trxA3* ORF
SD135B	GTAGACCGGGGCGAACTG	
SD348A	CGAACTCGGGCTGCTGTA	*trxB2* ORF
SD348B	CGCAACTGGTCCAAGGAG	
WQ053A	GCGGCATGAACGTCTACATAG	*mshA* ORF
WQ053B	GGGCTGAGTTCGACCGTG	
WQ046A	TCCCGTCGCCCGTATCTA	*mshC* ORF
WQ046B	GAACCTGCCGCTTGGTGT	
WQ045A	CTTCTTCCTGGCCGAGCG	*mshD* ORF
WQ045B	CACGTACACCTCGCCGATG	
SD252A	GTCCTTCCACCAGTTCCGTG	*sig22* ORF
SD252B	GGTTCGCGCTGCTTCTTG	
SD345A	AGCGGAATTCCTGGAAATG	*ectA* ORF
SD345B	AGGTCGAGGGTCTTGGAGTC	
SD147A	CACAAACGCTTCCCGAAC	*opuBA1* ORF
SD147B	GTCGTCTTACCGCAACCG	
SD146A	GCAGGTCAAGGACGAGAAAC	*opuBC1* ORF
SD146B	CCGGAGGACGACGAACAC	
SD149A	CATGTACAGGATGACCACTGG	*sav_5148* ORF
SD149B	CATCTCGAGCACTCCGATG	
SD387A	GTCGTCACTTATCGCAGCG	*opuBB2* ORF
SD387B	GGGATCAGCAGACCGATCAT	
SD344A	GCTCAGGTGCGTGACATC	*osaA* ORF
SD344B	CGGAGGAGAACGAGGACA	
SD191A	GATCCTCCTGGTCGATGAC	*osaB* ORF
SD191B	CAGTGCTTTGAGTGCTTCTTC	
SD160A	CGGTTCTCCACGGTGCTG	*katB* ORF
SD160B	CGCCCTCGCTGGTGTAGA	

*: Restriction endonuclease sites are underlined, and corresponding enzymes are shown in parentheses.

TABLE S2 | Putative targets of σ^8.

#	Accession number	Gene	Function	Score
**Regulatory function (179)**				
1	*SAV_29*		putative PadR-like family transcriptional regulator	7.9
2	*SAV_82*		putative TetR-family transcriptional regulator	6.9
3	*SAV_86*		putative erythropoiesis-stimulating protein	7.8
4	*SAV_143*		putative TetR-family transcriptional regulator	6.5
5	*SAV_205*		putative TetR-family transcriptional regulator	9.1
6	*SAV_213*	*sig60*	putative RNA polymerase ECF-subfamily sigma factor	8.1
7	*SAV_237*		putative DNA-binding protein	7
8	*SAV_306*		putative transcriptional regulatory protein	9
9	*SAV_323*		putative MerR-family transcriptional regulator	7.5
10	*SAV_431*		putative TetR-family transcriptional regulator	9.4
11	*SAV_436*		putative TetR-family transcriptional regulator	7.3
12	*SAV_458*	*hspR18_1*	putative hsp18 transcriptional regulator	8.3
13	*SAV_551*		putative TetR-family transcriptional regulator	7.2
14	*SAV_554*		putative LacI-family transcriptional regulator	8.6
15	*SAV_566*		putative TetR-family transcriptional regulator	6.6
16	*SAV_636*		putative AraC-family transcriptional regulator	9.1
17	*SAV_646*		putative two-component system sensor kinase	8.8
18	*SAV_669*		putative TetR-family transcriptional regulator	8.6
19	*SAV_675*		putative TetR-family transcriptional regulator	8.6
20	*SAV_691*	*hspR18_2*	putative hsp18 transcriptional regulator	7.7
21	*SAV_697*		putative MarR-family transcriptional regulator	8.5
22	*SAV_700*	*sig7*	putative RNA polymerase ECF-subfamily sigma factor	7.3
23	*SAV_722*		putative AraC-family transcriptional regulator	6.6
24	*SAV_736*		putative transcriptional regulator	8.5
25	*SAV_741*	*sig8*	putative RNA polymerase sigma factor	9.4
26	*SAV_775*		putative TetR-family transcriptional regulator	8.2
27	*SAV_786*	*prpJ3*	putative magnesium or manganese-dependent protein phosphatase	9.2
28	*SAV_792*		putative transcriptional regulator	10
29	*SAV_924*	*prpC4*	putative magnesium or manganese-dependent protein phosphatase	6.7
30	*SAV_980*		putative GntR-family transcriptional regulator	6.6
31	*SAV_1047*		putative regulatory protein	6.9
32	*SAV_1048*		putative DNA-binding protein	6.9
33	*SAV_1085*		putative two-component system sensor kinase	10.1
34	*SAV_1099*		putative MarR-family transcriptional regulator	6.5
35	*SAV_1100*		putative regulatory protein	6.5
36	*SAV_1103*		putative regulatory protein	8.2
37	*SAV_1148*	*popR*	putative DNA-binding protein	7.8

38	*SAV_1188*	*amiA*	putative MarR-family transcriptional regulator	6.7
39	*SAV_1271*		putative DNA-binding protein	7.6
40	*SAV_1356*		putative GntR-family transcriptional regulator	7.2
41	*SAV_1362*		putative transcriptional regulator	9
42	*SAV_1406*		putative GntR-family transcriptional regulator	9.4
43	*SAV_1693*	*whmD*	putative WhiB-family transcriptional regulator (wblH)	7.6
44	*SAV_1699*		putative MarR-family transcriptional regulator	7.2
45	*SAV_1742*		putative transcriptional regulator	8.2
46	*SAV_1845*		putative LysR-family transcriptional regulator	7.1
47	*SAV_1908*	*prpL2*	putative magnesium or manganese-dependent protein phosphatase	6.8
48	*SAV_1934*	*alkA1*	putative ADA-like regulatory protein	6.9
49	*SAV_1975*		putative DNA-binding protein	6.5
50	*SAV_1997*		putative IclR-family transcriptional regulator	9.1
51	*SAV_2012*		putative transcriptional regulator	8.5
52	*SAV_2033*		putative LuxR-family transcriptional regulator	7
53	*SAV_2042*		putative DNA-binding protein	6.9
54	*SAV_2091*		putative ROK-family transcriptional regulator	6.9
55	*SAV_2114*		putative LacI-family transcriptional regulator	7.7
56	*SAV_2124*		putative regulatory protein	7.6
57	*SAV_2175*		putative DNA-binding protein	7.4
58	*SAV_2179*	*cvnA4*	putative sensor-like histidine kinase	8.5
59	*SAV_2230*		putative two-component system response regulator	6.8
60	*SAV_2262*	*pkn5*	putative serine/threonine protein kinase	8.8
61	*SAV_2301*		putative regulatory protein	6.7
62	*SAV_2369*		putative regulatory protein	7.4
63	*SAV_2371*		putative two-component system response regulator	7.6
64	*SAV_2396*	*kdpD*	putative two-component system sensor kinase for high-affinity potassium transport system	7.8
65	*SAV_2419*		putative GntR-family transcriptional regulator	12
66	*SAV_2430*	*dcuS*	putative two-component system sensor kinase	7.5
67	*SAV_2462*	*nrdR*	putative regulatory protein	6.8
68	*SAV_2463*	*lexA*	putative SOS regulatory protein LexA	6.8
69	*SAV_2511*	*osaB*	putative two-component system response regulator (response to osmoadaptation and osmotic stress)	8.6
70	*SAV_2512*	*osaA*	putative two-component system sensor kinase (response to osmoadaptation and osmotic stress)	7.1
71	*SAV_2601*		putative PadR-like family transcriptional regulator	7.5
72	*SAV_2677*		putative AsnC-family transcriptional regulator	6.5
73	*SAV_2687*		putative IclR-family transcriptional regulator	7.5
74	*SAV_2694*	*cvnA6*	putative sensor-like histidine kinase	7.5
75	*SAV_2759*		putative TetR-family transcriptional regulator	7.2
76	*SAV_2785*		putative regulatory protein	10
77	*SAV_2814*		putative TetR-family transcriptional regulator	7

78	*SAV_2831*		putative TetR-family transcriptional regulator	9
79	*SAV_2853*		putative MarR-family transcriptional regulator	8.3
80	*SAV_2961*	*cvnB8*	hypothetical protein	6.7
81	*SAV_2964*		putative MarR-family transcriptional regulator	8.7
82	*SAV_3012*	*rsbW1*	putative anti-sigma factor	7.5
83	*SAV_3016*	*wblE*	putative WhiB-family transcriptional regulator; putative role in cell cycle control	7
84	*SAV_3038*	*sig22*	putative RNA polymerase ECF-subfamily sigma factor	13
85	*SAV_3053*	*catR*	putative hydrogen peroxide sensitive repressor	7.5
86	*SAV_3186*		putative regulatory protein	8.7
87	*SAV_3203*		putative LuxR-family transcriptional regulator	7.9
88	*SAV_3231*	*oxyR*	putative hydrogen peroxide sensing regulator, LysR-family transcriptional regulator	9.4
89	*SAV_3352*	*smrA*	putative two-component system response regulator	7
90	*SAV_3488*		putative regulator protein containing histidine kinase domain	7.7
91	*SAV_3489*		putative anti-sigma factor antagonist	7.7
92	*SAV_3490*	*sig29*	putative RNA polymerase sigma factor	7.7
93	*SAV_3546*		putative TetR-family transcriptional regulator	7
94	*SAV_3571*		putative MarR-family transcriptional regulator	8.2
95	*SAV_3690*	*fruR*	putative DeoR-family transcriptional regulator	9.9
96	*SAV_3693*		putative TetR-family transcriptional regulator	7.8
97	*SAV_3694*	*sig30*	putative RNA polymerase sigma factor	7
98	*SAV_3837*		putative regulatory protein	7.4
99	*SAV_3843*		putative anti-sigma factor antagonist	8.9
100	*SAV_3844*	*sig31*	putative RNA polymerase ECF-subfamily sigma factor	8.9
101	*SAV_3877*	*cvnA9*	putative sensor-like histidine kinase	8.1
102	*SAV_3888*	*sig32*	putative RNA polymerase ECF-subfamily sigma factor	6.7
103	*SAV_3895*		putative MarR-family transcriptional regulator	6.8
104	*SAV_3923*		putative RpiR-family transcriptional regulator	7.3
105	*SAV_3940*		putative ROK-family transcriptional regulator	7.5
106	*SAV_3970*		putative CarD-like transcriptional regulator	6.7
107	*SAV_4009*	*prpG1*	putative magnesium or manganese-dependent protein phosphatase	8.2
108	*SAV_4042*		putative two-component system response regulator	7.8
109	*SAV_4086*		putative regulatory protein	6.6
110	*SAV_4087*		putative DNA-binding protein	6.6
111	*SAV_4130*	*bldC*	putative MerR-family transcriptional regulator	9.2
112	*SAV_4159*		putative two-component system response regulator	6.8
113	*SAV_4186*	*sig35*	putative RNA polymerase sigma factor	9.6
114	*SAV_4193*		putative TetR-family transcriptional regulator	8.5
115	*SAV_4263*	*prpA2*	putative serine/threonine protein phosphatase	8.7
116	*SAV_4295*		putative PadR-like family transcriptional regulator	8.3
117	*SAV_4398*		putative regulatory protein	8
118	*SAV_4406*		putative LysR-family transcriptional regulator	7.9

119	*SAV_4421*		putative regulatory protein	7.9
120	*SAV_4427*	*sig39*	putative RNA polymerase ECF-subfamily sigma factor	6.6
121	*SAV_4773*	*abaB2*	putative LysR-family transcriptional regulator	6.8
122	*SAV_4530*		putative TetR-family transcriptional regulator	8.4
123	*SAV_4592*		putative transcriptional regulator with cyclic nucleotide-binding domain	7.1
124	*SAV_4647*		putative regulatory protein	7.2
125	*SAV_4649*		putative DNA-binding protein	7.3
126	*SAV_4683*		putative ArsR-family transcriptional regulator	6.5
127	*SAV_4701*		putative TetR-family transcriptional regulator	6.5
128	*SAV_4734*	*rsbN*	putative anti-sigma factor	8.4
129	*SAV_4758*		putative transcriptional regulator	8.4
130	*SAV_4762*		putative AraC-family transcriptional regulator	7.6
131	*SAV_4767*		putative two-component system response regulator	8.1
132	*SAV_4857*		putative GntR-family transcriptional regulator	6.5
133	*SAV_4875*		putative transcriptional regulator	7.6
134	*SAV_4997*	*wblB*	putative WhiB-family transcriptional regulator; putative role in cell cycle control	7.4
135	*SAV_4998*	*whiK*	putative two-component system response regulator (bldM)	7.4
136	*SAV_5006*	*pkn19*	putative serine/threonine protein kinase	6.9
137	*SAV_5013*		putative DNA-binding protein	9.4
138	*SAV_5014*		putative regulatory protein	9.4
139	*SAV_5148*		putative AsnC-family transcriptional regulator	7.4
140	*SAV_5237*		putative sensory box/GGDEF family protein	9.3
141	*SAV_5261*	*bdpA*	AraC-family transcriptional regulator	8
142	*SAV_5312*		putative LacI-family transcriptional regulator	7.5
143	*SAV_5320*	*rbsR*	putative LacI-family transcriptional regulator, ribose operon repressor	7.4
144	*SAV_5336*		putative MarR-family transcriptional regulator	8.4
145	*SAV_5389*		putative PadR-like family transcriptional regulator	6.8
146	*SAV_5429*	*prpB11*	putative magnesium or manganese-dependent protein phosphatase	6.9
147	*SAV_5491*	*comEA*	putative exogenous DNA-binding protein	7.1
148	*SAV_5579*		putative IclR-family transcriptional regulator	8.6
149	*SAV_5626*		putative DNA-binding protein	10
150	*SAV_5700*		putative transcriptional regulator with cyclic nucleotide-binding domain	6.6
151	*SAV_5775*		putative MarR-family transcriptional regulator	6.7
152	*SAV_5854*		putative TetR-family transcriptional regulator	9
153	*SAV_5869*		putative two-component system sensor kinase	6.8
154	*SAV_5976*	*malR1*	putative repressor of malE transcription	6.8
155	*SAV_6019*		putative DeoR-family transcriptional regulator	7.4
156	*SAV_6198*		putative TetR-family transcriptional regulator	6.6
157	*SAV_6253*		putative DNA-binding protein	7.1

158	*SAV_6365*		putative GntR-family transcriptional regulator	9.7
159	*SAV_6427*		putative transcriptional regulator	7.2
160	*SAV_6465*		putative GntR-family transcriptional regulator	7.7
161	*SAV_6544*		putative regulatory protein	6.7
162	*SAV_6599*		putative TetR-family transcriptional regulator	7.5
163	*SAV_6630*	*idnR*	putative GntR-family transcriptional regulator	6.9
164	*SAV_6666*	*gylR*	putative glycerol operon regulatory protein, IclR-family	7.3
165	*SAV_6702*	*cvnA10*	putative sensor-like histidine kinase	9.3
166	*SAV_6710*		putative PadR-like family transcriptional regulator	8
167	*SAV_6724*		putative GntR-family transcriptional regulator	9
168	*SAV_6752*		putative aminotransferase	7.6
169	*SAV_6799*	*pkn30*	putative serine/threonine protein kinase	8.3
170	*SAV_6954*		putative IclR-family transcriptional regulator	6.7
171	*SAV_7002*		putative LysR-family transcriptional regulator	7
172	*SAV_7041*		putative transcriptional regulatory protein	9.8
173	*SAV_7046*		putative TetR-family transcriptional regulator	8
174	*SAV_7090*	*lpsR*	putative lpsA1 transcriptional activator	7.8
175	*SAV_7156*		putative LacI-family transcriptional regulator	7.6
176	*SAV_7278*		putative GntR-family transcriptional regulator	6.6
177	*SAV_7346*		putative LacI-family transcriptional regulator	9.9
178	*SAV_7393*		putative regulatory protein	8.2
179	*SAV_7452*		putative anti-sigma factor antagonist	7.1
**Secondary metabolism (10)**				
180	*SAV_840*		putative thioesterase	7.5
181	*SAV_1654*	*hopE*	squalene/phytoene synthase	8
182	*SAV_2368*	*pks5*	putative modular polyketide synthase	7.4
183	*SAV_2373*	*pks9-1*	putative acyl carrier protein	7.1
184	*SAV_2563*	*dxr*	putative 1-deoxy-D-xylulose 5-phosphate reductoisomerase	8
185	*SAV_2836*	*sppG*	putative WhiE I homolog	8.9
186	*SAV_3032*	*ezs*	epi-isozizaene synthase (sesquiterpene cyclase)	7.1
187	*SAV_3202*		putative thioesterase	7.9
188	*SAV_3665*	*pks8-8*	putative 3-oxoacyl-ACP synthase I	7.3
189	*SAV_7362*	*pks1-3*	putative modular polyketide synthase	8
**Metabolism of amino acids & related molecules (33)**				
190	*SAV_801*	*argG1*	putative argininosuccinate synthase	6.9
191	*SAV_1257*	*paaK*	putative phenylacetate:CoA ligase	6.5
192	*SAV_1819*	*ilvB3*	putative TPP-requiring enzyme (acetolactate synthase II)	7.2
193	*SAV_1938*	*ggt1*	putative gamma-glutamyltranspeptidase	7.6
194	*SAV_2111*	*mmuM*	putative homocysteine S-methyltransferase (S-methylmethionine-dependent)	9.4
195	*SAV_2579*		putative aldehyde dehydrogenase	7.7
196	*SAV_2592*		putative aldehyde dehydrogenase	8.7
197	*SAV_2686*	*leuC*	putative 3-isopropylmalate dehydratase subunit large	7.5
198	*SAV_2716*	*ureAB*	putative urease beta/gamma subunit	10.1

199	*SAV_2723*	*rocA*	putative delta-1-pyrroline-5-carboxylate dehydrogenase	6.5
200	*SAV_2918*	*thrA*	putative homoserine dehydrogenase	7.6
201	*SAV_3125*	*dapE*	putative succinyl-diaminopimelate desuccinylase	7.7
202	*SAV_3496*	*hutU*	putative urocanate hydratase	6.6
203	*SAV_3562*	*prsA1*	putative ribose-phosphate pyrophosphokinase	9.1
204	*SAV_3934*	*thrC3*	putative threonine synthase	7.4
205	*SAV_4132*	*vdh*	putative valine dehydrogenase (NADP+)	7.1
206	*SAV_4517*	*dapC*	putative N-succinyldiaminopimelate aminotransferase	8.6
207	*SAV_4519*	*speE*	putative spermidine synthase	7.3
208	*SAV_4872*	*lap*	putative leucyl aminopeptidase, secreted (aminopeptidase S)	6.7
209	*SAV_4963*	*glmS1*	putative L-glutamine-D-fructose-6-phosphate amidotransferase	7.2
210	*SAV_5488*	*leuS*	putative leucyl-tRNA synthetase	6.9
211	*SAV_5580*	*dapA3*	putative dihydrodipicolinate synthase	8.6
212	*SAV_5765*		putative aminotransferase	9.1
213	*SAV_6005*	*glnA3*	putative glutamine synthetase	8.4
214	*SAV_6189*	*gltB*	putative glutamate synthase(NADPH) large subunit	8.9
215	*SAV_6360*		putative 5-dehydro-4-deoxyglucarate dehydratase	7.7
216	*SAV_6507*	*ald*	putative L-alanine dehydrogenase	7.8
217	*SAV_6667*	*metH*	putative 5-methyltetrahydrofolate:homocysteine S-methyltransferase	7.3
218	*SAV_6725*	*glnA4*	putative glutamine synthetase	9
219	*SAV_6763*	*argC*	putative N-acetyl-gamma-glutamyl-phosphate reductase	6.6
220	*SAV_6955*	*hcaC2*	putative ferredoxin subunit of phenylpropionate dioxygenase	6.7
221	*SAV_7162*	*cysK3*	putative cysteine synthase	8.2
222	*SAV_7345*	*amdA2*	putative amidase	9.9
**Carbohydrate Metabolism (33)**				
223	*SAV_372*	*suhB2*	putative myo-inositol 1-monophosphatase	7.3
224	*SAV_628*	*agaA1*	putative alpha-galactosidase, secreted	8
225	*SAV_1325*	*bga2*	putative beta-galactosidase	8.1
226	*SAV_1514*	*maeA1*	putative malate dehydrogenase (oxaloacetate-decarboxylating; NAD-requiring)	8
227	*SAV_1662*	*galE6*	putative UDP-glucose 4-epimerase	7.6
228	*SAV_1700*	*pcaI*	putative 3-oxoadipate succinyl-CoA transferase alpha subunit	7.2
229	*SAV_1764*	*lamA1*	putative endo-1,3-beta-glucanase, secreted	9.8
230	*SAV_1818*	*sucC1*	putative succinyl-CoA synthetase beta subunit	7.2
231	*SAV_2000*	*aceB1*	putative malate synthase	6.7
232	*SAV_2024*	*gip*	putative hydroxypyruvate isomerase	8.2
233	*SAV_2428*		putative citrate synthase-like protein	8.9
234	*SAV_2747*	*citA3*	putative citrate synthase	8.9
235	*SAV_2972*	*sucA*	putative 2-oxoglutarate dehydrogenase	7.8
236	*SAV_3018*	*gamA*	putative glucosamine-6-phosphate isomerase	7.8
237	*SAV_3258*	*chiA2*	putative chitinase A, secreted	7.5
238	*SAV_3576*	*galT*	putative galactose-1-phosphate uridylyltransferase	8.7
239	*SAV_3870*	*maeA2*	putative malate dehydrogenase (oxaloacetate-decarboxylating;	10.8

			NAD-requiring)	
240	*SAV_3942*	*fruK*	putative tagatose-6-phosphate kinase	7.7
241	*SAV_4318*	*gnd2*	putative 6-phosphogluconate dehydrogenase	7.9
242	*SAV_4599*	*acsA1*	putative acetyl-CoA synthetase	7.4
243	*SAV_5126*	*maeB2*	putative malate dehydrogenase (oxaloacetate-decarboxylating; NADP-requiring)	10.4
244	*SAV_5151*	*glcD2*	putative (S)-2-hydroxy-acid oxidase	7.8
245	*SAV_5217*	*cslZ*	putative endo-1,4-beta-glucanase, secreted	6.8
246	*SAV_5743*	*abfA*	putative alpha-L-arabinofuranosidase	7.3
247	*SAV_5981*	*amyA4*	putative secreted alpha-amylase	7.9
248	*SAV_6020*	*aceE2*	putative pyruvate dehydrogenase E1 component	6.9
249	*SAV_6217*	*pykA2*	putative pyruvate kinase	6.9
250	*SAV_6314*	*tal2*	putative transaldolase	8.8
251	*SAV_6315*	*tkt2*	putative transketolase	6.5
252	*SAV_6375*		putative secreted pectate lyase	7.2
253	*SAV_6629*	*idnK*	putative gluconokinase	6.9
254	*SAV_7150*	*iolC2*	putative 5-dehydro-2-deoxygluconokinase	8.3
255	*SAV_7348*	*lpdB*	putative dihydrolipoamide dehydrogenase	10.4
**Fatty acid and lipid metabolism (22)**				
256	*SAV_1104*	*fadD3*	putative acyl-CoA synthetase, long-chain fatty acid:CoA ligase	8.2
257	*SAV_1591*	*fadS1*	putative fatty acid desaturase	7.1
258	*SAV_1691*	*desA*	putative fatty acid desaturase	8.4
259	*SAV_1917*	*pssA*	putative phosphatidylserine synthase	7.1
260	*SAV_2291*	*fabC3*	putative acyl carrier protein	6.5
261	*SAV_2388*		putative phosphopantetheinyl transferase	7.2
262	*SAV_3120*		putative enoyl-CoA hydratase/isomerase	6.8
263	*SAV_3461*		putative lipase	7.7
264	*SAV_3512*	*fadA2*	putative 3-ketoacyl-CoA thiolase/acetyl-CoA acetyltransferase	6.6
265	*SAV_3807*	*ltp2*	putative nonspecific lipid-transfer protein	9.6
266	*SAV_4206*	*fadD9*	putative acyl-CoA synthetase, long-chain fatty acid:CoA ligase	7.6
267	*SAV_4224*	*glpQ2*	putative glycerophosphoryl diester phosphodiesterase	7
268	*SAV_4390*	*fadE24*	putative acyl-CoA dehydrogenase	8.1
269	*SAV_4531*		putative lipase/esterase	8.4
270	*SAV_5003*	*glpD2*	putative glycerol-3-phosphate dehydrogenase	7.1
271	*SAV_6136*	*fabG6*	putative 3-oxoacyl-ACP reductase	9.5
272	*SAV_6271*	*glpQ6*	putative glycerophosphoryl diester phosphodiesterase	8.4
273	*SAV_6428*	*echA14*	putative enoyl-CoA hydratase/isomerase	6.8
274	*SAV_6559*	*lpsA2*	putative secreted lipase	8.1
275	*SAV_6600*	*fadE3*	putative acyl-CoA dehydrogenase	7.5
276	*SAV_6826*	*pgsA2*	putative phosphatidylglycerophosphate synthase	6.7
277	*SAV_7089*	*lpsA1*	putative secreted lipase	7.8
**Nucleotide metabolism (2)**				
278	*SAV_3445*	*purN*	putative phosphoribosylglycinamide formyltransferase	6.5
279	*SAV_6522*	*cmk*	putative cytidylate kinase	6.8

**Protein synthesis, folding and modification (30)**				
280	*SAV_786*	*prpJ3*	putative magnesium or manganese-dependent protein phosphatase	9.2
281	*SAV_1117*		putative metallopeptidase, secreted	7.2
282	*SAV_1231*		putative secreted tripeptidyl-peptidase C	8.2
283	*SAV_1974*	*map2*	putative methionyl aminopeptidase	6.5
284	*SAV_2443*		putative trypsin-like protease, secreted	11.6
285	*SAV_2558*	*proS1*	putative prolyl-tRNA synthetase	8.7
286	*SAV_2626*	*tsf*	putative elongation factor EF-Ts	8.6
287	*SAV_2913*	*rpmE2*	putative ribosomal protein L31	9
288	*SAV_3223*	*prpE2*	putative magnesium or manganese-dependent protein phosphatase	7.9
289	*SAV_3462*		putative zoocin A (family M23 peptidase)	8.5
290	*SAV_4152*		putative metallopeptidase, secreted	8.8
291	*SAV_4244*	*serS1*	putative seryl-tRNA synthetase	7.8
292	*SAV_4289*	*rpsF*	putative ribosomal protein S6	6.7
293	*SAV_4407*		putative snapalysin (secreted metalloprotease)	7.9
294	*SAV_4664*	*tilS*	putative tRNA(Ile)-lysidine synthase	8.1
295	*SAV_4700*		putative zoocin A	7.3
296	*SAV_4748*	*argS2*	putative arginyl-tRNA synthetase	7.6
297	*SAV_4749*	*lysS1*	putative lysyl-tRNA synthetase	7.6
298	*SAV_4910*	*rplK*	putative ribosomal protein L11	7.8
299	*SAV_4912*	*rplJ*	putative ribosomal protein L10	6.5
300	*SAV_4917*	*rpsL*	ribosomal protein S12	8.3
301	*SAV_4949*	*infA1*	putative translation initiation factor IF-1	7.2
302	*SAV_4951*	*rpsM*	putative ribosomal protein S13	6.6
303	*SAV_5155*		putative protease, secreted	8.8
304	*SAV_5159*	*clpS*	putative ATP-dependent Clp protease adaptor protein	9.6
305	*SAV_6233*	*rpsA*	putative ribosomal protein S1	8.5
306	*SAV_6451*		putative tripeptidyl-peptidase S, secreted	7.4
307	*SAV_6634*		putative peptidase	7.1
308	*SAV_6658*	*eif2*	putative initiation factor eIF-2B alpha subunit	8
309	*SAV_7101*	*clpP3*	putative ATP-dependent Clp protease proteolytic subunit 2	6.7
**DNA synthesis, repair, recombination, modification and packaging (20)**				
310	*SAV_226*		putative reverse transcriptase homolog; similar to GII intron	7
311	*SAV_367*	*helZ1*	putative SNF2/RAD54 family helicase	7.1
312	*SAV_497*	*int3*	putative recombinase/integrase	8.9
313	*SAV_2684*	*hupB*	putative histone-like DNA-binding protein	7.6
314	*SAV_2915*	*rho1*	putative transcription termination factor Rho	7.1
315	*SAV_3542*	*savM1*	putative type II restriction-modification system DNA adenine-specific methylase	7.1
316	*SAV_3735*	*pra1*	putative replication activator protein Pra	7.8
317	*SAV_3988*	*pra2*	putative replication activator protein Pra	7.2
318	*SAV_4016*	*helD2*	putative ATP-dependent DNA helicase	7

319	*SAV_4284*	*dnaB*	putative replicative DNA helicase	10.3
320	*SAV_4285*	*dinF*	putative DNA-damage-inducible protein F	10.3
321	*SAV_4288*	*ssb1*	putative single-stranded DNA-binding protein	8.9
322	*SAV_4317*	*dnaN1*	putative DNA polymerase III beta subunit	6.8
323	*SAV_4593*	*nth*	putative endonuclease III	7.1
324	*SAV_5202*		putative ATP-dependent helicase	7.9
325	*SAV_6143*	*dnaE1*	putative DNA polymerase III alpha subunit	7.3
326	*SAV_6513*		putative DNA hydrolase	6.5
327	*SAV_6555*	*dnaE2*	putative DNA polymerase III alpha subunit	7.7
328	*SAV_6875*	*priA*	putative primosomal protein	6.5
329	*SAV_7571*	*ttrA2*	putative helicase	9.4
**RNA synthesis and modification (6)**				
330	*SAV_2231*	*rnd*	putative ribonuclease D	7.8
331	*SAV_2475*	*miaA*	putative delta(2)-isopentenylpyrophosphate tRNA-adenosine transferase	6.9
332	*SAV_3301*	*greA*	putative transcription elongation factor	7.8
333	*SAV_4914*	*rpoB*	RNA polymerase beta subunit	9.8
334	*SAV_5289*		putative secreted ribonuclease	7.7
335	*SAV_7054*		putative ATP-dependent RNA helicase	7.2
**Membrane bioenergetics (10)**				
336	*SAV_1171*	*cyp5*	putative cytochrome P450	6.5
337	*SAV_1507*	*fprB*	putative NAD(P)H-ferredoxin reductase	9
338	*SAV_1941*	*fdh*	putative NAD-dependent formate dehydrogenase	9.4
339	*SAV_2888*	*atpI*	putative ATP synthase protein I	7.5
340	*SAV_3536*	*cyp16*	putative cytochrome P450	7
341	*SAV_4260*	*cydA1*	putative cytochrome bd-I oxidase subunit I (cytochrome bd complex)	9.5
342	*SAV_4276*	*fhbA*	putative flavohemoprotein	8.7
343	*SAV_5953*	*fhbB*	putative flavohemoprotein	8.3
344	*SAV_6097*	*fprE*	putative NAD(P)H-ferredoxin reductase	9.9
345	*SAV_6113*	*cynT3*	putative carbonic anhydrase	8.4
**Metabolism of cofactors and vitamins (20)**				
346	*SAV_1482*	*thiX1*	putative flavin-dependent reductase	9.2
347	*SAV_2676*	*thiL*	putative thiamine monophosphate kinase	6.5
348	*SAV_3330*	*birA*	putative biotin apoprotein ligase	6.9
349	*SAV_3442*	*folD1*	putative methylenetetrahydrofolate dehydrogenase and methenyltetrahydrofolate cyclohydrolase	6.8
350	*SAV_3517*	*pobA*	putative p-hydroxybenzoate hydroxylase	6.6
351	*SAV_3828*		putative pimeloyl-CoA synthetase	7.1
352	*SAV_3945*	*thiX2*	putative flavin-dependent reductase	8.8
353	*SAV_4040*	*moaD*	putative molybdopterin converting factor	6.5
354	*SAV_4121*	*ribA3*	putative GTP cyclohydrolase II	8
355	*SAV_4811*	*ubiD*	putative 3-octaprenyl-4-hydroxybenzoate carboxy-lyase	8.1
356	*SAV_4813*	*cofH1*	putative 7,8-didemethyl-8-hydroxy-5-deazariboflavin synthase	8.5

			subunit 2	
357	*SAV_5158*	*pncB*	putative nicotinate phosphoribosyltransferase	9.6
358	*SAV_5930*	*hmuO*	putative heme oxygenase	6.6
359	*SAV_5943*	*panB*	putative 3-methyl-2-oxobutanoate hydroxymethyltransferase	7.2
360	*SAV_6099*	*thiE*	putative thiamine-phosphate pyrophosphorylase	7.2
361	*SAV_6139*	*phoD1*	putative alkaline phosphatase, secreted	8
362	*SAV_6794*	*cobL2*	putative precorrin-6Y C5,15-methyltransferase	7.2
363	*SAV_6796*	*cysG*	putative uroporphyrin-III methyltransferase	6.8
364	*SAV_7093*	*bioB*	putative biotin synthase	7.2
365	*SAV_7094*	*bioF*	putative 8-amino-7-oxononanoate synthase	7.2
**Detoxification and adaptation to atypical conditions (22)**				
366	*SAV_348*	*katB*	putative catalase	7.6
367	*SAV_459*	*hsp18_1*	putative heat shock protein	8.3
368	*SAV_692*	*hsp18_2*	putative heat shock protein	7.7
369	*SAV_1149*		putative secreted esterase	7.8
370	*SAV_1531*	*nos*	putative nitric oxide synthase	6.5
371	*SAV_2113*		putative carboxylesterase, secreted	7.7
372	*SAV_2830*	*trxA6*	putative thioredoxin	9
373	*SAV_3138*	*mshA*	putative glycosyltransferase (forming 1D-myo-inositÿl 2-acetamido-2-deoxy-alpha-D-glucopyranoside)	7.4
374	*SAV_3675*	*pacB1*	putative penicillin acylase, secreted	7.4
375	*SAV_3695*	*bar*	putative phosphinothricin N-acetyltransferase	7
376	*SAV_3932*	*cspB2*	putative cold shock protein	6.8
377	*SAV_4057*	*mshD*	putative N-cysteinyl-1-D-myo-inosityl-2-amino-2-deoxy-alpha-D-gluco pyranoside acetyltransferase	7.1
378	*SAV_4484*	*dnaK1*	putative heat shock protein Hsp70	8.6
379	*SAV_4776*	*cspD5*	putative cold shock protein	6.5
380	*SAV_4820*	*cspD6*	putative cold shock protein	8.6
381	*SAV_4991*	*groES1*	putative GroES	7.6
382	*SAV_5159*	*clpS*	putative ATP-dependent Clp protease adaptor protein	9.6
383	*SAV_5764*	*blaA4*	putative beta-lactamase	7.7
384	*SAV_5999*	*arsC*	putative arsenate reductase	6.9
385	*SAV_6003*	*htpX2*	putative heat shock protein, protease	6.6
386	*SAV_7243*	*trxB2*	putative thioredoxin reductase	7.4
387	*SAV_7347*	*trxA3*	putative thioredoxin	10.4
**Cell wall and cell envelope (9)**				
388	*SAV_1319*	*bacA1*	putative undecaprenyl-diphosphatase	6.8
389	*SAV_2784*		putative polysaccharide deacetylase	7.4
390	*SAV_2965*		putative lysozyme precursor	8.7
391	*SAV_3225*	*pbp2*	putative penicillin-binding protein	9.5
392	*SAV_3561*	*glmU*	putative UDP-N-acetylglucosamine pyrophosphorylase	8
393	*SAV_3781*	*dacC*	putative D-alanyl-D-alanine carboxypeptidase	8.9
394	*SAV_4294*	*pbp5*	putative penicillin-binding protein	8.3

395	*SAV_7219*	*pbp12*	putative penicillin-binding protein	7.5
396	*SAV_7297*		putative oligosaccharide deacetylase, secreted	6.6
**Cell division and differentiation (7)**				
397	*SAV_2528*		putative FtsK/SpoIIIE family protein	8.8
398	*SAV_4666*	*ftsH*	putative cell division protein FtsH	9.8
399	*SAV_5104*	*ftsE*	putative cell division ATP-binding protein	7.9
400	*SAV_5469*	*obg*	putative GTP-binding protein	6.8
401	*SAV_6124*	*ftsZ*	putative cell division GTPase FtsZ	6.5
402	*SAV_6508*	*parA2*	putative partitioning or sporulation protein	8.5
403	*SAV_6810*	*ssgC*	putative cell division protein (probably reverse function of SsgA)	8.3
**Transport & binding proteins (75)**				
404	*SAV_560*	*nicT1*	putative high-affinity nickel-transport protein	9.2
405	*SAV_791*		putative cytosine/uracil/thiamine/allantoin permease	10
406	*SAV_794*		putative amino acid transporter	7.3
407	*SAV_877*	*cpt*	putative chloramphenicol 3-O phosphotransferase	7.7
408	*SAV_1028*		putative multiple sugar ABC transporter substrate-binding protein	7.5
409	*SAV_1125*	*potD3*	putative polyamine ABC transporter substrate-binding protein	7.1
410	*SAV_1133*		putative membrane transport protein	6.8
411	*SAV_1326*		putative ABC transporter substrate-binding protein	8.1
412	*SAV_1342*		putative transmembrane efflux protein	6.5
413	*SAV_1947*		putative cytosine/uracil/thiamine/allantoin permease	7.4
414	*SAV_2013*		putative transmembrane efflux protein	8.5
415	*SAV_2178*	*opuBC1*	putative ABC transporter substrate-binding protein	8.5
416	*SAV_2187*		putative ABC transporter substrate-binding protein	7.4
417	*SAV_2275*		putative transmembrane efflux protein	6.5
418	*SAV_2390*		putative amino acid permease	8.8
419	*SAV_2391*	*trkA*	putative potassium transporter	8.8
420	*SAV_2432*	*ssuA4*	putative ABC transporter substrate-binding protein	6.8
421	*SAV_2577*	*lplA2*	putative multiple sugar ABC transporter substrate-binding protein	8.5
422	*SAV_2603*		putative multi-drug efflux transporter	7.3
423	*SAV_2613*		putative ABC transporter ATP-binding protein	7.3
424	*SAV_2657*	*araE*	putative L-arabinose permease	8.5
425	*SAV_2769*	*oppC1*	putative peptide ABC transporter permease protein	8.4
426	*SAV_2813*		putative transmembrane efflux protein	7
427	*SAV_2862*		putative ABC transporter ATP-binding protein	7.1
428	*SAV_3044*	*sugE*	putative SMR-type multi-drug efflux transporter	8.2
429	*SAV_3090*	*oppA3*	putative peptide ABC transporter substrate-binding protein	6.7
430	*SAV_3107*	*corA2*	putative metal-transport protein	7.7
431	*SAV_3149*	*oppA4*	putative peptide ABC transporter substrate-binding protein	8.9
432	*SAV_3150*	*bldKA1*	putative peptide ABC transporter permease protein	8.9
433	*SAV_3176*	*bldKA2*	putative peptide ABC transporter permease protein	7.6

434	*SAV_3277*	*gntP*	putative low-affinity gluconate transporter	7
435	*SAV_3370*		putative simple sugar ABC transporter substrate-binding protein	7.1
436	*SAV_3505*	*opuBA1*	putative ABC transporter ATP-binding protein	6.8
437	*SAV_3552*		putative integral membrane transport protein	7
438	*SAV_3577*		putative Na+/galactose cotransporter	8.7
439	*SAV_3606*	*cbiM*	putative cobalt ABC transporter permease protein	6.7
440	*SAV_3634*		putative ABC transporter ATP-binding protein	6.6
441	*SAV_3676*		putative sodium/proton antiporter	7.4
442	*SAV_3681*	*mscL*	putative mechanosensitive channel	7.4
443	*SAV_3698*		putative transmembrane efflux protein	7
444	*SAV_3848*		putative lipoprotein	9.1
445	*SAV_3852*		putative ABC transporter ATP-binding protein	7
446	*SAV_4477*		putative integrin-like protein	7.1
447	*SAV_4488*		putative simple sugar ABC transporter substrate-binding protein	7
448	*SAV_4702*		putative transmembrane efflux protein	6.5
449	*SAV_4721*		putative transmembrane sulfate transport protein	8.2
450	*SAV_4722*		putative ABC transporter ATP-binding protein	8.2
451	*SAV_4904*		putative transmembrane efflux protein	7
452	*SAV_5098*		putative multiple sugar ABC transporter substrate-binding protein	6.7
453	*SAV_5131*	*dasA2*	putative multiple sugar ABC transporter substrate-binding protein	6.6
454	*SAV_5168*	*ptsC2*	putative phosphotransferase system IIC component	7
455	*SAV_5169*	*ptsB*	putative phosphotransferase system IIB component	7
456	*SAV_5256*	*cebE*	putative cellobiose ABC transporter substrate-binding protein	6.5
457	*SAV_5302*	*nagZ5*	putative beta-N-acetylhexosaminidase, secreted	6.6
458	*SAV_5343*		putative amino acid transporter	8.1
459	*SAV_5419*	*ssuA6*	putative ABC transporter substrate-binding protein	7.5
460	*SAV_5619*	*oppA8*	putative peptide ABC transporter substrate-binding protein	9.9
461	*SAV_5681*		putative ABC transporter ATP-binding protein	8.1
462	*SAV_5865*		putative membrane transport protein	8.7
463	*SAV_5926*		putative lipoprotein	8
464	*SAV_5944*		putative transmembrane efflux protein	7.2
465	*SAV_5977*	*malE*	putative maltose-binding protein	6.8
466	*SAV_6264*	*corA1*	putative metal-transport protein	7.5
467	*SAV_6276*		putative integral membrane export protein	6.9
468	*SAV_6419*	*pitH2*	putative low-affinity inorganic phosphate transporter	7
469	*SAV_6527*		putative peptidoglycan-binding protein, secreted	9.4
470	*SAV_6657*		putative simple sugar ABC transporter substrate-binding protein	8
471	*SAV_6665*	*glpF1*	putative glycerol uptake facilitator protein	6.8
472	*SAV_6709*		putative amino acid permease	8
473	*SAV_6711*		putative ABC transporter ATP-binding protein	7.2
474	*SAV_6812*		putative multiple sugar ABC transporter substrate-binding protein	7.7

475	*SAV_6975*	*ptsA*	putative phosphoenolpyruvate-dependent sugar phosphotransferase	7.8
476	*SAV_7038*		putative siderophore binding protein	7
477	*SAV_7210*	*gltI3*	putative polar amino acid ABC transporter substrate-binding protein	8.2
478	*SAV_7294*		putative amino acid transporter protein	7.6
**Gas vesicle (1)**				
479	*SAV_1890*	*gvpO2*	putative gas vesicle synthesis protein	6.5
**Mobile and extrachromosomal element functions (8)**				
480	*SAV_118*		putative IS5 family IS1647-like transposase	7.6
481	*SAV_229*		putative transposase	7
482	*SAV_254*		putative IS5 family ISFa13B-like transposase	8.4
483	*SAV_260*		putative transposase	7
484	*SAV_300*		putative IS256 family IS1164-like transposase	7.6
485	*SAV_473*		putative ISL3 family IS469-like transposase	8.7
486	*SAV_670*	*insA*	putative IS701 family ISFsp9-like transposase	6.8
487	*SAV_7558*		putative IS5 family IS493-like transposase	6.9
**Unknown or unclassified genes (453)**				
488	*SAV_1*		hypothetical protein	6.5
489	*SAV_14*		putative membrane protein	6.6
490	*SAV_27*		hypothetical protein	7.7
491	*SAV_40*		hypothetical protein	6.9
492	*SAV_41*		putative secreted protein	6.6
493	*SAV_44*		putative unsaturated glucuronyl hydrolase	8.5
494	*SAV_52*		hypothetical protein	7.1
495	*SAV_69*		hypothetical protein	6.8
496	*SAV_75*		putative endoribonuclease L-PSP	7.7
497	*SAV_83*		hypothetical protein	6.9
498	*SAV_87*		hypothetical protein	7.8
499	*SAV_96*		hypothetical protein	7
500	*SAV_107*		hypothetical protein	6.6
501	*SAV_123*		putative O-methyltransferase	6.8
502	*SAV_124*		putative membrane protein	7
503	*SAV_125*		putative membrane protein	7
504	*SAV_203*		putative secreted protein	7.2
505	*SAV_204*		putative membrane protein	9.1
506	*SAV_211*		hypothetical protein	6.6
507	*SAV_222*		hypothetical protein	7.5
508	*SAV_228*		hypothetical protein	6.8
509	*SAV_241*		hypothetical protein	8.3
510	*SAV_242*		putative secreted protein	7.4
511	*SAV_249*		putative secreted protein	7.1
512	*SAV_253*		putative MutT-family protein	7.2
513	*SAV_264*		hypothetical protein	10.1

514	*SAV_275*		hypothetical protein	7.2
515	*SAV_346*		putative secreted protein	7.7
516	*SAV_362*		putative secreted protein	9
517	*SAV_366*		hypothetical protein	7.1
518	*SAV_369*		hypothetical protein	6.6
519	*SAV_402*		hypothetical protein	8.3
520	*SAV_422*		hypothetical protein	6.7
521	*SAV_425*		putative oxidoreductase	8
522	*SAV_426*		hypothetical protein	6.8
523	*SAV_432*		putative secreted protein	9.4
524	*SAV_442*		hypothetical protein	6.7
525	*SAV_466*		putative secreted protein	10.1
526	*SAV_478*		hypothetical protein	10.5
527	*SAV_485*		hypothetical protein	7.1
528	*SAV_505*		putative monooxygenase	9.4
529	*SAV_539*		hypothetical protein	8.2
530	*SAV_550*		hypothetical protein	7.4
531	*SAV_555*	*celA1*	putative secreted endo-1,4-beta-glucanase	10.4
532	*SAV_559*		putative secreted protein	7.4
533	*SAV_571*		hypothetical protein	8.5
534	*SAV_620*		putative hydrolase	8.7
535	*SAV_633*		putative secreted protein	6.9
536	*SAV_640*		hypothetical protein	9.9
537	*SAV_641*		hypothetical protein	7.4
538	*SAV_650*		putative methyltransferase	7.5
539	*SAV_658*		putative membrane protein	6.6
540	*SAV_666*		hypothetical protein	6.7
541	*SAV_668*		putative lyase	8.6
542	*SAV_671*		hypothetical protein	6.8
543	*SAV_676*		putative dehydrogenase	8.6
544	*SAV_696*		hypothetical protein	8.5
545	*SAV_702*		putative acyltransferase	7.8
546	*SAV_714*		putative phosphatase	8.4
547	*SAV_725*		hypothetical protein	7.2
548	*SAV_732*		hypothetical protein	6.9
549	*SAV_740*		hypothetical protein	6.8
550	*SAV_747*		hypothetical protein	7.1
551	*SAV_748*		putative secreted protein	6.6
552	*SAV_768*		hypothetical protein	7.1
553	*SAV_769*		hypothetical protein	6.8
554	*SAV_774*		putative monooxygenase	8.2
555	*SAV_777*		hypothetical protein	10.7
556	*SAV_778*		hypothetical protein	7.7
557	*SAV_781*		putative secreted protein	6.6

558	*SAV_783*		hypothetical protein	6.9
559	*SAV_796*		hypothetical protein	7.9
560	*SAV_805*		hypothetical protein	7.3
561	*SAV_806*		hypothetical protein	7.3
562	*SAV_811*		hypothetical protein	6.7
563	*SAV_830*		hypothetical protein	7.5
564	*SAV_858*		hypothetical protein	6.8
565	*SAV_876*		hypothetical protein	8.5
566	*SAV_892*		hypothetical protein	9.1
567	*SAV_894*		hypothetical protein	6.6
568	*SAV_895*		hypothetical protein	6.8
569	*SAV_903*		putative membrane protein	8.7
570	*SAV_907*		hypothetical protein	9.7
571	*SAV_908*		putative dehydrogenase	9.7
572	*SAV_920*		putative secreted protein	7.5
573	*SAV_923*		putative hydrolase	6.7
574	*SAV_925*		hypothetical protein	11.3
575	*SAV_967*		putative glycosyl hydrolase, secreted	6.8
576	*SAV_983*	*gmt*	geranyl diphosphate methyltransferase	8.6
577	*SAV_991*		hypothetical protein	6.6
578	*SAV_1039*		hypothetical protein	8.4
579	*SAV_1062*		hypothetical protein	7.5
580	*SAV_1084*		hypothetical protein	7.3
581	*SAV_1089*		hypothetical protein	6.9
582	*SAV_1109*		hypothetical protein	7.9
583	*SAV_1124*		hypothetical protein	7.1
584	*SAV_1168*		hypothetical protein	7.3
585	*SAV_1179*		hypothetical protein	8.6
586	*SAV_1182*		hypothetical protein	6.6
587	*SAV_1201*		putative dehydrogenase	7.9
588	*SAV_1230*		putative secreted protein	8.2
589	*SAV_1238*		putative amidase	7.2
590	*SAV_1256*		putative family S33 peptidase	6.5
591	*SAV_1330*		hypothetical protein	7.2
592	*SAV_1355*		putative hydrolase	7.2
593	*SAV_1363*		putative secreted protein	9
594	*SAV_1385*		hypothetical protein	7.5
595	*SAV_1408*		putative racemase	8.9
596	*SAV_1428*		hypothetical protein	6.6
597	*SAV_1430*		putative membrane protein	6.7
598	*SAV_1434*		hypothetical protein	6.7
599	*SAV_1460*		putative ATP/GTP-binding protein	7.9
600	*SAV_1487*		hypothetical protein	6.7
601	*SAV_1490*		hypothetical protein	8.8

602	*SAV_1506*		hypothetical protein	9
603	*SAV_1543*		putative secreted protein	6.5
604	*SAV_1571*		hypothetical protein	6.9
605	*SAV_1585*		hypothetical protein	6.9
606	*SAV_1638*		hypothetical protein	9.3
607	*SAV_1694*		putative hydroxylase	7.6
608	*SAV_1741*		hypothetical protein	8.2
609	*SAV_1746*		putative hydrolase	6.9
610	*SAV_1747*		hypothetical protein	6.9
611	*SAV_1749*		putative phosphoesterase (SimX4 homolog)	9.3
612	*SAV_1750*		hypothetical protein	7.6
613	*SAV_1762*		hypothetical protein	8.1
614	*SAV_1763*		putative lipoprotein	8.1
615	*SAV_1764*	*lamA1*	putative endo-1,3-beta-glucanase, secreted	9.8
616	*SAV_1796*		hypothetical protein	8.3
617	*SAV_1807*		putative NLP/P60-family secreted protein (PgpA peptidase)	8
618	*SAV_1844*		putative transmembrane protein	7.1
619	*SAV_1849*		putative oxidoreductase	6.6
620	*SAV_1851*		putative dehydrogenase	6.5
621	*SAV_1852*		hypothetical protein	7
622	*SAV_1869*		putative integral membrane protein	7
623	*SAV_1921*		putative integral membrane protein	7.7
624	*SAV_1926*		hypothetical protein	7
625	*SAV_1927*		putative integral membrane protein	7
626	*SAV_1939*		hypothetical protein	7.6
627	*SAV_1942*	*crcB3*	putative camphor resistance protein CrcB	9.4
628	*SAV_1976*		hypothetical protein	7.7
629	*SAV_2022*		hypothetical protein	7.1
630	*SAV_2023*		putative secreted protein	7.1
631	*SAV_2027*		hypothetical protein	6.9
632	*SAV_2050*		hypothetical protein	6.8
633	*SAV_2051*		putative hydrolase	7.1
634	*SAV_2052*		hypothetical protein	7.1
635	*SAV_2058*		hypothetical protein	7.1
636	*SAV_2065*		hypothetical protein	7.2
637	*SAV_2071*		hypothetical protein	7.5
638	*SAV_2087*		putative cellulose-binding protein	6.8
639	*SAV_2104*		hypothetical protein	7.5
640	*SAV_2105*	*ephB*	putative epoxide hydrolase	7.5
641	*SAV_2107*		putative secreted protein	9.2
642	*SAV_2108*		putative rhamnogalacturonase B precursor, secreted	9.2
643	*SAV_2122*		putative integral membrane protein, ribonuclease BN-like family	7.3
644	*SAV_2149*		putative copper amine oxidase, secreted	7

645	*SAV_2170*	putative cholesterol esterase	6.9
646	*SAV_2174*	hypothetical protein	7.4
647	*SAV_2199*	hypothetical protein	8.4
648	*SAV_2200*	hypothetical protein	8.4
649	*SAV_2219*	putative secreted protein	6.7
650	*SAV_2253*	putative alpha-1,2-mannosidase, secreted	6.9
651	*SAV_2299*	hypothetical protein	7.7
652	*SAV_2326*	hypothetical protein	8.4
653	*SAV_2331*	putative membrane protein	7.6
654	*SAV_2346*	putative secreted protein	6.6
655	*SAV_2359*	hypothetical protein	7.1
656	*SAV_2403*	hypothetical protein	8
657	*SAV_2425*	putative secreted protein	7.5
658	*SAV_2451*	putative secreted protein	9.6
659	*SAV_2476*	hypothetical protein	6.9
660	*SAV_2514*	putative aminotransferase	12.1
661	*SAV_2527*	putative membrane protein	8.8
662	*SAV_2564*	putative secreted protein	8
663	*SAV_2566*	hypothetical protein	7.3
664	*SAV_2570*	putative glycosyl hydrolase	7.1
665	*SAV_2593*	hypothetical protein	8.7
666	*SAV_2615*	hypothetical protein	8.9
667	*SAV_2645*	hypothetical protein	8.3
668	*SAV_2707*	putative secreted protein	7.9
669	*SAV_2720*	hypothetical protein	9.3
670	*SAV_2755*	putative multicopper oxidase, secreted	11.7
671	*SAV_2758*	putative membrane protein	7.2
672	*SAV_2910*	hypothetical protein	6.6
673	*SAV_2941*	hypothetical protein	9.8
674	*SAV_2953*	hypothetical protein	8.7
675	*SAV_2954*	hypothetical protein	8.7
676	*SAV_3055*	putative integral membrane protein	6.6
677	*SAV_3056*	hypothetical protein	6.6
678	*SAV_3058*	putative secreted protein	8.3
679	*SAV_3071*	hypothetical protein	7.5
680	*SAV_3101*	hypothetical protein	6.5
681	*SAV_3104*	hypothetical protein	8.5
682	*SAV_3126*	hypothetical protein	7.7
683	*SAV_3163*	hypothetical protein	11
684	*SAV_3188*	hypothetical protein	7.1
685	*SAV_3194*	putative hydrolase	7.1
686	*SAV_3207*	putative GTP binding protein	7.6
687	*SAV_3208*	hypothetical protein	7.6
688	*SAV_3219*	hypothetical protein	8.8

689	*SAV_3226*	putative integral membrane protein	9.5
690	*SAV_3271*	hypothetical protein	6.8
691	*SAV_3272*	hypothetical protein	6.8
692	*SAV_3288*	hypothetical protein	7.1
693	*SAV_3323*	hypothetical protein	10.2
694	*SAV_3324*	hypothetical protein	10.2
695	*SAV_3356*	hypothetical protein	8.4
696	*SAV_3364*	hypothetical protein	7.4
697	*SAV_3446*	hypothetical protein	6.5
698	*SAV_3457*	putative MoxR-like ATPase	9.6
699	*SAV_3458*	hypothetical protein	9.6
700	*SAV_3464*	putative NLP/P60-family secreted protein (PgpA peptidase)	7.8
701	*SAV_3511*	putative secreted protein	6.8
702	*SAV_3513*	putative secreted protein	7.2
703	*SAV_3535*	putative secreted protein	7
704	*SAV_3539*	hypothetical protein	9.9
705	*SAV_3545*	hypothetical protein	7
706	*SAV_3555*	hypothetical protein	7.5
707	*SAV_3570*	putative trans-aconitate methyltransferase	8.2
708	*SAV_3580*	putative secreted protein	8.3
709	*SAV_3583*	putative membrane protein	8.1
710	*SAV_3686*	hypothetical protein	6.9
711	*SAV_3747*	hypothetical protein	9.9
712	*SAV_3767*	putative secreted protein	10.8
713	*SAV_3782*	hypothetical protein	8.9
714	*SAV_3800*	hypothetical protein	7.7
715	*SAV_3819*	hypothetical protein	7.5
716	*SAV_3847*	hypothetical protein	9.1
717	*SAV_3869*	hypothetical protein	7.8
718	*SAV_3876*	putative oxidoreductase	8.1
719	*SAV_3884*	putative secreted protein	8.2
720	*SAV_3896*	hypothetical protein	6.8
721	*SAV_3903*	putative integral membrane protein	7.6
722	*SAV_3922*	hypothetical protein	7.3
723	*SAV_3996*	hypothetical protein	8.6
724	*SAV_4008*	putative NLP/P60-family secreted protein (PgpA peptidase)	8.3
725	*SAV_4019*	hypothetical protein	7.5
726	*SAV_4041*	putative hydrolase	7.8
727	*SAV_4055*	hypothetical protein	6.9
728	*SAV_4077*	putative Pit accessory protein	6.8
729	*SAV_4078*	hypothetical protein	6.8
730	*SAV_4093*	hypothetical protein	8.1
731	*SAV_4117*	putative oxidoreductase	7.3
732	*SAV_4125*	hypothetical protein	6.8

733	*SAV_4131*		hypothetical protein	7.1
734	*SAV_4148*		putative membrane protein	9.4
735	*SAV_4160*		putative secreted protein	6.8
736	*SAV_4164*		putative membrane protein	7.6
737	*SAV_4166*		putative membrane protein	7.8
738	*SAV_4177*		putative secreted protein	7
739	*SAV_4179*		hypothetical protein	6.6
740	*SAV_4228*		hypothetical protein	6.5
741	*SAV_4270*		hypothetical protein	7.8
742	*SAV_4276*	*fhbA*	putative flavohemoprotein	8.7
743	*SAV_4290*		putative membrane protein	6.7
744	*SAV_4291*		hypothetical protein	6.5
745	*SAV_4302*		hypothetical protein	7.3
746	*SAV_4343*		hypothetical protein	9.3
747	*SAV_4385*		hypothetical protein	6.9
748	*SAV_4386*		hypothetical protein	6.9
749	*SAV_4391*		hypothetical protein	8.1
750	*SAV_4392*		putative pirin-like protein	6.7
751	*SAV_4393*		putative integral membrane protein	6.8
752	*SAV_4402*		hypothetical protein	7.3
753	*SAV_4404*		hypothetical protein	8.9
754	*SAV_4405*		hypothetical protein	8.9
755	*SAV_4420*		putative secreted protein	7.9
756	*SAV_4438*		putative secreted protein	9.6
757	*SAV_4449*		hypothetical protein	8.2
758	*SAV_4450*		hypothetical protein	8.2
759	*SAV_4458*		putative secreted protein	9
760	*SAV_4461*		putative secreted protein	10.6
761	*SAV_4482*		putative iron-sulfur binding reductase	7.1
762	*SAV_4483*		putative glycosyltransferase, secreted	7.1
763	*SAV_4494*		hypothetical protein	6.5
764	*SAV_4512*		hypothetical protein	7
765	*SAV_4525*		hypothetical protein	7
766	*SAV_4532*		putative membrane protein	6.7
767	*SAV_4555*		hypothetical protein	11
768	*SAV_4556*		hypothetical protein	11
769	*SAV_4578*		hypothetical protein	8.3
770	*SAV_4633*		hypothetical protein	8.6
771	*SAV_4673*		putative membrane protein	6.9
772	*SAV_4674*		putative membrane protein	6.9
773	*SAV_4675*		putative secreted protein	7.3
774	*SAV_4695*		putative Lsr2-like protein	7.7
775	*SAV_4696*		putative proline-rich protein	6.7
776	*SAV_4714*		hypothetical protein	7.3

777	*SAV_4731*		hypothetical protein	6.6
778	*SAV_4752*		putative secreted protein	8.2
779	*SAV_4756*		hypothetical protein	6.9
780	*SAV_4766*		putative glycosyltransferase	8.1
781	*SAV_4807*		putative acetyltransferase	7.5
782	*SAV_4815*		putative membrane protein	6.7
783	*SAV_4827*		putative sugar hydrolase, secreted	8.6
784	*SAV_4828*		putative membrane protein	8.6
785	*SAV_4873*		hypothetical protein	7.5
786	*SAV_4874*		hypothetical protein	7.6
787	*SAV_4893*		hypothetical protein	7.1
788	*SAV_4984*	*xynA2*	putative endo-1,4-beta xylanase, secreted	8.8
789	*SAV_5018*		putative secreted protein	9.8
790	*SAV_5041*	*cdo2*	putative cysteine dioxygenase	7.2
791	*SAV_5103*		putative secreted protein	6.8
792	*SAV_5118*		hypothetical protein	6.9
793	*SAV_5129*		putative secreted protein	7.2
794	*SAV_5152*		putative membrane protein	7.1
795	*SAV_5163*		hypothetical protein	6.8
796	*SAV_5176*	*bcp2*	putative bacterioferritin comigratory protein	8.6
797	*SAV_5177*		putative membrane protein	8.6
798	*SAV_5187*		putative membrane protein	7.9
799	*SAV_5209*	*hpcE*	putative 2-hydroxyhepta-2,4-diene-1,7-dioate isomerase	7.5
800	*SAV_5213*		hypothetical protein	8.5
801	*SAV_5238*		putative secreted protein	9.3
802	*SAV_5321*		hypothetical protein	7.4
803	*SAV_5322*		hypothetical protein	8.4
804	*SAV_5349*		putative secreted protein	7.3
805	*SAV_5351*		putative secreted protein	6.7
806	*SAV_5363*		possible ATP-binding protein	8
807	*SAV_5390*		hypothetical protein	6.8
808	*SAV_5406*		hypothetical protein	8
809	*SAV_5428*		hypothetical protein	6.9
810	*SAV_5432*		hypothetical protein	7.9
811	*SAV_5441*		hypothetical protein	7
812	*SAV_5494*		hypothetical protein	7
813	*SAV_5543*		hypothetical protein	6.7
814	*SAV_5547*		hypothetical protein	7.7
815	*SAV_5554*		hypothetical protein	7.9
816	*SAV_5591*		putative secreted protein	6.9
817	*SAV_5652*		hypothetical protein	7
818	*SAV_5666*		putative secreted protein	7.7
819	*SAV_5670*		hypothetical protein	6.7
820	*SAV_5672*		putative integral membrane protein	6.5

821	*SAV_5692*		hypothetical protein	6.9
822	*SAV_5693*		hypothetical protein	6.9
823	*SAV_5695*		hypothetical protein	8.8
824	*SAV_5744*		hypothetical protein	6.5
825	*SAV_5751*		hypothetical protein	8
826	*SAV_5772*		putative dehydratase	7.2
827	*SAV_5773*		hypothetical protein	7.2
828	*SAV_5828*		putative secreted protein	8.9
829	*SAV_5829*		putative hydrolase	6.5
830	*SAV_5855*	*wbpY*	putative glycosyltransferase	9
831	*SAV_5870*	*ufaA*	putative MaoC-like dehydratase	6.8
832	*SAV_5876*		hypothetical protein	6.6
833	*SAV_5946*		putative membrane protein	7.3
834	*SAV_5952*		hypothetical protein	8.3
835	*SAV_5955*		hypothetical protein	7.9
836	*SAV_5956*		hypothetical protein	7.9
837	*SAV_5990*		putative secreted protein	7.5
838	*SAV_5993*		putative reductase	8.2
839	*SAV_6000*		hypothetical protein	6.9
840	*SAV_6004*		putative secreted protein	6.6
841	*SAV_6006*		putative membrane protein	8.4
842	*SAV_6008*		hypothetical protein	6.8
843	*SAV_6071*		hypothetical protein	7
844	*SAV_6073*		hypothetical protein	7.7
845	*SAV_6087*		hypothetical protein	6.6
846	*SAV_6114*	*mraW*	putative S-adenosylmethionine-dependent methyltransferase	8.4
847	*SAV_6142*		hypothetical protein	7.3
848	*SAV_6144*		hypothetical protein	9.1
849	*SAV_6197*		putative dehydrogenase	7.8
850	*SAV_6232*		putative methyltransferase	8.5
851	*SAV_6246*		putative secreted protein	7.9
852	*SAV_6250*		hypothetical protein	6.6
853	*SAV_6255*		hypothetical protein	7.5
854	*SAV_6263*		hypothetical protein	7.3
855	*SAV_6265*		putative methyltransferase	7.5
856	*SAV_6272*		putative integral membrane protein	8.4
857	*SAV_6317*		hypothetical protein	6.6
858	*SAV_6332*		putative alpha-L-arabinofuranosidase	9.2
859	*SAV_6359*		hypothetical protein	7.7
860	*SAV_6374*		putative secreted rhamnogalacturonan acetylesterase	7.3
861	*SAV_6382*		putative secreted pectate lyase	6.5
862	*SAV_6423*		hypothetical protein	7.5
863	*SAV_6456*		hypothetical protein	10.9
864	*SAV_6473*		putative secreted protein	10.2

865	*SAV_6478*		putative secreted protein	6.9
866	*SAV_6479*		putative secreted protein	6.8
867	*SAV_6482*		putative secreted protein	7.5
868	*SAV_6489*		hypothetical protein	6.9
869	*SAV_6490*		putative hydrolase	6.9
870	*SAV_6526*		hypothetical protein	9.4
871	*SAV_6531*		putative secreted protein	7.2
872	*SAV_6532*		putative secreted protein	7.2
873	*SAV_6539*		putative integral membrane protein	7.1
874	*SAV_6560*	*cpbD2*	putative chitin-binding protein, secreted	7.4
875	*SAV_6561*		putative acetyltransferase	7.4
876	*SAV_6562*		hypothetical protein	6.6
877	*SAV_6563*		hypothetical protein	6.6
878	*SAV_6584*		hypothetical protein	8.1
879	*SAV_6593*		putative secreted protein	8.8
880	*SAV_6610*		putative secreted protein	6.8
881	*SAV_6611*		putative dehydrogenase	6.8
882	*SAV_6624*		putative oxidoreductase	9
883	*SAV_6668*		putative hydrolase, secreted	7.5
884	*SAV_6672*		hypothetical protein	9
885	*SAV_6751*		putative integral membrane protein	7.6
886	*SAV_6753*		putative acetyltransferase	7.9
887	*SAV_6755*		hypothetical protein	7.9
888	*SAV_6756*		putative alpha-L-arabinofuranosidase I	7.9
889	*SAV_6811*		putative membrane protein	8.3
890	*SAV_6817*		putative integral membrane protein	6.6
891	*SAV_6818*		hypothetical protein	6.6
892	*SAV_6898*		putative secreted protein	7.8
893	*SAV_6912*		hypothetical protein	6.8
894	*SAV_6913*		hypothetical protein	6.8
895	*SAV_6931*		hypothetical protein	6.5
896	*SAV_6943*		hypothetical protein	6.7
897	*SAV_6967*		hypothetical protein	7.2
898	*SAV_6968*		putative MutT-like protein	7.2
899	*SAV_6984*		hypothetical protein	7.5
900	*SAV_6988*		hypothetical protein	6.7
901	*SAV_6989*		hypothetical protein	7.3
902	*SAV_7001*		hypothetical protein	7
903	*SAV_7032*		putative membrane protein	7.1
904	*SAV_7036*		hypothetical protein	6.9
905	*SAV_7039*		putative sugar acetyltransferase	7
906	*SAV_7040*		putative membrane protein	9.8
907	*SAV_7045*		putative reductase	8
908	*SAV_7095*		hypothetical protein	7.3

909	*SAV_7102*		hypothetical protein	6.7
910	*SAV_7121*		putative secreted protein	7.4
911	*SAV_7155*		putative oxidoreductase	7.6
912	*SAV_7157*		hypothetical protein	7.6
913	*SAV_7173*		hypothetical protein	7.4
914	*SAV_7193*	*ppx3*	putative exopolyphosphatase	7.9
915	*SAV_7194*		putative methyltransferase	7.9
916	*SAV_7227*		hypothetical protein	7
917	*SAV_7232*		hypothetical protein	9.9
918	*SAV_7268*		putative exo-alpha-galactosidase	7.9
919	*SAV_7290*		putative membrane protein	6.6
920	*SAV_7291*		hypothetical protein	6.9
921	*SAV_7298*		putative membrane protein	6.7
922	*SAV_7299*		putative glycine-rich protein	6.7
923	*SAV_7327*		hypothetical protein	9.4
924	*SAV_7344*		putative membrane protein	6.6
925	*SAV_7385*		hypothetical protein	7.6
926	*SAV_7394*		hypothetical protein	8.2
927	*SAV_7414*	*srlD*	putative sorbitol-6-phosphate 2-dehydrogenase	8.3
928	*SAV_7434*		hypothetical protein	7.3
929	*SAV_7437*		hypothetical protein	6.6
930	*SAV_7444*	*mrpF*	putative membrane protein	7.9
931	*SAV_7458*		hypothetical protein	7.1
932	*SAV_7459*		putative membrane-associated oxidoreductase	7.1
933	*SAV_7465*		hypothetical protein	6.6
934	*SAV_7466*		hypothetical protein	6.9
935	*SAV_7515*		hypothetical protein	7.5
936	*SAV_7518*		hypothetical protein	6.6
937	*SAV_7524*		hypothetical protein	7.6
938	*SAV_7562*		hypothetical protein	8.3
939	*SAV_7565*		hypothetical protein	9.1
940	*SAV_7572*		hypothetical protein	9.4
**Total: 940**				

Yell-highlighted numbers: σ^8 targets confirmed by EMSAs.

Underlined numbers: putative targets confirmed by EMSAs as not bound by σ^8.
